# Supplementary material for: Foraging in a non-foraging task: Fitness maximization explains human risk preference dynamics under changing environment
Source: PLoS Comput Biol. 2024 May 13;20(5):e1012080. doi: 10.1371/journal.pcbi.1012080 (PMC11115364; doi:10.1371/journal.pcbi.1012080)
Supplement: S1 Table — Integrated Bayesian information criterion (iBIC) of models with the sigmoid and approach-avoidance choice rules were computed and compared. A lower iBIC value indicates a better model performance. (PDF) [file pcbi.1012080.s001.pdf]

Choice rule	Experiment	Model description								
		Risk-return	Multiplicative				Additive			
			$m \times p$	$m \times \pi(p)$	$v(m) \times p$	$v(m) \times \pi(p)$	$m + p$	$m + \pi(p)$	$v(m) + p$	$v(m) + \pi(p)$
Sigmoid	Experiment 1	12705	17478	17345	13526	11399	15324	11346	14456	11124
	Experiment 2	21191	30257	30037	23124	19273	26427	19192	24851	18711
Approach-avoidance	Experiment 1	12661	17149	16679	13284	11521	15295	11419	14429	11216
	Experiment 2	21217	29716	29387	22747	19480	26415	19260	24786	18940
