# Supplementary material for: Foraging in a non-foraging task: Fitness maximization explains human risk preference dynamics under changing environment
Source: PLoS Comput Biol. 2024 May 13;20(5):e1012080. doi: 10.1371/journal.pcbi.1012080 (PMC11115364; doi:10.1371/journal.pcbi.1012080)
Supplement: S2 Table — (PDF) [file pcbi.1012080.s002.pdf]

Model	Parameter	Group	First environment		Second environment	
			Mean	SD	Mean	SD
Risk-return model	α	PR	1.49E-02	6.81E-03	1.69E-02	9.82E-03
		RP	1.77E-02	8.61E-03	1.93E-02	1.06E-02
Multiplicative model	γ	PR	4.07	2.07	3.10	2.02
		RP	2.65	1.59	2.84	1.76
	λ	PR	0.434	0.128	0.444	0.231
		RP	0.454	0.221	0.389	0.191
Additive model	γ	PR	5.00	1.76	3.98	2.23
		RP	3.61	2.01	3.68	1.73
	λ	PR	0.260	0.026	0.308	0.053
		RP	0.291	0.045	0.266	0.054
	wp	PR	0.665	0.067	0.741	0.071
		RP	0.720	0.063	0.714	0.068

Model	Parameter	Group	First environment		Second environment		Third environment	
			Mean	SD	Mean	SD	Mean	SD
Risk-return model	α	IPI	1.76E-02	6.79E-03	1.70E-02	7.25E-03	1.67E-02	8.14E-03
		IRI	1.69E-02	8.09E-03	1.87E-02	1.13E-02	1.79E-02	1.22E-02
Multiplicative model	γ	IPI	3.56	1.67	3.21	1.62	2.59	1.38
		IRI	3.84	2.19	2.92	1.86	2.61	1.87
	λ	IPI	0.394	0.171	0.430	0.227	0.471	0.217
		IRI	0.404	0.178	0.419	0.237	0.461	0.262
Additive model	γ	IPI	4.64	2.03	4.21	1.87	3.50	1.70
		IRI	4.75	1.94	3.69	2.00	3.20	1.70
	λ	IPI	0.253	0.036	0.285	0.050	0.320	0.048
		IRI	0.263	0.044	0.275	0.053	0.300	0.058
	wp	IPI	0.690	0.068	0.720	0.069	0.744	0.057
		IRI	0.695	0.066	0.714	0.080	0.724	0.080

Model	Parameter	Group	Environment	Group	Environment	df	*t*	*p*	Significance
Risk-return model	α	PR	Poor	RP	Rich	242	-2.76	0.006	**
		PR	Poor + Rich	RP	Poor + Rich	242	-2.36	0.019	*
Multiplicative model	γ	PR	Poor	RP	Rich	242	6.06	0.000	***
		PR	Poor + Rich	RP	Poor + Rich	242	4.07	0.000	***
	λ	PR	Poor	RP	Rich	242	-0.82	1.000	n.s
		PR	Poor + Rich	RP	Poor + Rich	242	0.75	0.912	n.s
Additive model	γ	PR	Poor	RP	Rich	242	5.68	0.000	***
		PR	Poor + Rich	RP	Poor + Rich	242	3.82	0.001	***
	λ	PR	Poor	RP	Rich	242	-6.19	1.000	n.s
		PR	Poor + Rich	RP	Poor + Rich	242	1.07	0.855	n.s
	wp	PR	Poor	RP	Rich	242	-6.62	0.000	***
		PR	Poor + Rich	RP	Poor + Rich	242	-1.81	0.217	n.s

Model	Parameter	Group	Environment	Group	Environment	df	*t*	*p*	Significance
Risk-return model	α	IPI	Poor - 1st int.	IRI	Rich - 1st int.	280	-3.67	0.000	***
		IPI	2nd int - 1st int.	IRI	2nd int- 1st int.	280	-2.35	0.019	*
Multiplicative model	γ	IPI	Poor - 1st int.	IRI	Rich - 1st int.	280	2.67	0.016	*
		IPI	2nd int - 1st int.	IRI	2nd int- 1st int.	280	1.14	0.507	n.s
	λ	IPI	Poor - 1st int.	IRI	Rich - 1st int.	280	1.31	0.379	n.s
		IPI	2nd int - 1st int.	IRI	2nd int- 1st int.	280	1.04	0.595	n.s
Additive model	γ	IPI	Poor - 1st int.	IRI	Rich - 1st int.	280	3.10	0.006	**
		IPI	2nd int - 1st int.	IRI	2nd int- 1st int.	280	1.83	0.205	n.s
	λ	IPI	Poor - 1st int.	IRI	Rich - 1st int.	280	5.19	0.000	***
		IPI	2nd int - 1st int.	IRI	2nd int- 1st int.	280	6.65	0.000	***
	wp	IPI	Poor - 1st int.	IRI	Rich - 1st int.	280	1.87	1.000	n.s
		IPI	2nd int - 1st int.	IRI	2nd int- 1st int.	280	4.01	1.000	n.s
