# Supplementary material for: Foraging in a non-foraging task: Fitness maximization explains human risk preference dynamics under changing environment
Source: PLoS Comput Biol. 2024 May 13;20(5):e1012080. doi: 10.1371/journal.pcbi.1012080 (PMC11115364; doi:10.1371/journal.pcbi.1012080)
Supplement: S3 Table — (PDF) [file pcbi.1012080.s003.pdf]

Model	Parameter	Group	First environment		Second environment	
			Mean	SD	Mean	SD
Risk-return model	α	PR	1.70E-02	5.29E-03	1.81E-02	1.05E-02
		RP	1.85E-02	9.20E-03	2.10E-02	1.13E-02
	η	PR	0.150	0.124	0.061	0.026
		RP	0.051	0.015	0.070	0.029
Multiplicative model	γ	PR	3.20	1.22	2.89	1.88
		RP	2.54	1.52	2.60	1.36
	λ	PR	0.439	0.154	0.444	0.231
		RP	0.451	0.212	0.384	0.212
	η	PR	0.096	0.055	0.044	0.014
		RP	0.026	0.003	0.058	0.024
Additive model	γ	PR	4.67	1.56	3.88	2.24
		RP	3.60	1.98	3.55	1.65
	λ	PR	0.312	0.035	0.341	0.062
		RP	0.320	0.051	0.289	0.051
	wp	PR	0.744	0.056	0.779	0.055
		RP	0.760	0.056	0.749	0.063
	η	PR	0.067	0.019	0.030	0.004
		RP	0.028	0.004	0.036	0.006

Model	Parameter	Group	Environment	Group	Environment	df	*t*	*p*	Significance
Risk-return model	α	PR	Poor	RP	Rich	242	-1.52	0.259	n.s
		PR	Poor + Rich	RP	Poor + Rich	242	-1.95	0.105	n.s
	η	PR	Poor	RP	Rich	242	9.20	0.000	***
		PR	Poor + Rich	RP	Poor + Rich	242	7.39	0.000	***
Multiplicative model	γ	PR	Poor	RP	Rich	242	3.66	0.001	***
		PR	Poor + Rich	RP	Poor + Rich	242	2.76	0.019	*
	λ	PR	Poor	RP	Rich	242	-0.49	1.000	n.s
		PR	Poor + Rich	RP	Poor + Rich	242	0.97	1.000	n.s
	η	PR	Poor	RP	Rich	242	14.73	0.000	***
		PR	Poor + Rich	RP	Poor + Rich	242	10.00	0.000	***
Additive model	γ	PR	Poor	RP	Rich	242	4.60	0.000	***
		PR	Poor + Rich	RP	Poor + Rich	242	3.25	0.005	**
	λ	PR	Poor	RP	Rich	242	-1.43	0.923	n.s
		PR	Poor + Rich	RP	Poor + Rich	242	3.61	0.000	***
	wp	PR	Poor	RP	Rich	242	-2.31	0.011	*
		PR	Poor + Rich	RP	Poor + Rich	242	0.95	0.828	n.s
	η	PR	Poor	RP	Rich	242	23.31	0.000	***
		PR	Poor + Rich	RP	Poor + Rich	242	17.64	0.000	***

Model	Parameter	Group	First environment		Second environment		Third environment	
			Mean	SD	Mean	SD	Mean	SD
Risk-return model	α	IPI	1.88E-02	6.33E-03	1.78E-02	7.33E-03	1.75E-02	8.45E-03
		IRI	1.87E-02	8.70E-03	1.95E-02	1.19E-02	1.85E-02	1.22E-02
	η	IPI	0.092	0.055	0.057	0.023	0.055	0.021
		IRI	0.117	0.058	0.038	0.008	0.031	0.005
Multiplicative model	γ	IPI	3.19	1.53	2.91	1.42	2.43	1.31
		IRI	3.61	1.93	2.71	1.65	2.35	1.44
	λ	IPI	0.381	0.193	0.421	0.231	0.472	0.218
		IRI	0.396	0.188	0.409	0.240	0.454	0.261
	η	IPI	0.094	0.108	0.069	0.089	0.038	0.014
		IRI	0.056	0.014	0.058	0.041	0.061	0.062
Additive model	γ	IPI	4.56	1.97	4.23	1.92	3.51	1.81
		IRI	4.63	1.88	3.80	2.14	3.25	1.78
	λ	IPI	0.283	0.043	0.312	0.060	0.352	0.060
		IRI	0.293	0.046	0.310	0.067	0.333	0.072
	wp	IPI	0.743	0.061	0.766	0.058	0.784	0.050
		IRI	0.746	0.065	0.768	0.068	0.766	0.069
	η	IPI	0.053	0.012	0.050	0.025	0.042	0.018
		IRI	0.055	0.011	0.044	0.017	0.031	0.009

Model	Parameter	Group	Environment	Group	Environment	df	*t*	*p*	Significance
Risk-return model	α	IPI	Poor - 1st int.	IRI	Rich - 1st int.	280	-2.61	0.019	*
		IPI	2nd int - 1st int.	IRI	2nd int- 1st int.	280	-1.20	0.462	n.s
	η	IPI	Poor - 1st int.	IRI	Rich - 1st int.	280	6.98	0.000	***
		IRI	2nd int - 1st int.	IRI	2nd int- 1st int.	280	7.32	0.000	***
Multiplicative model	γ	IPI	Poor - 1st int.	IRI	Rich - 1st int.	280	3.21	0.004	**
		IPI	2nd int - 1st int.	IRI	2nd int- 1st int.	280	2.50	0.039	*
	λ	IPI	Poor - 1st int.	IRI	Rich - 1st int.	280	1.54	0.499	n.s
		IPI	2nd int - 1st int.	IRI	2nd int- 1st int.	280	1.62	0.428	n.s
	η	IPI	Poor - 1st int.	IRI	Rich - 1st int.	280	-2.70	1.000	n.s
		IRI	2nd int - 1st int.	IRI	2nd int- 1st int.	280	-6.05	1.000	n.s
Additive model	γ	IPI	Poor - 1st int.	IRI	Rich - 1st int.	280	2.41	0.066	n.s
		IPI	2nd int - 1st int.	IRI	2nd int- 1st int.	280	1.45	0.591	n.s
	λ	IPI	Poor - 1st int.	IRI	Rich - 1st int.	280	2.68	0.004	**
		IPI	2nd int - 1st int.	IRI	2nd int- 1st int.	280	5.42	0.000	***
	wp	IPI	Poor - 1st int.	IRI	Rich - 1st int.	280	0.28	0.609	n.s
		IPI	2nd int - 1st int.	IRI	2nd int- 1st int.	280	3.80	1.000	n.s
	η	IPI	Poor - 1st int.	IRI	Rich - 1st int.	280	3.14	0.001	***
		IRI	2nd int - 1st int.	IRI	2nd int- 1st int.	280	7.11	0.000	***
