# Supplementary material for: Foraging in a non-foraging task: Fitness maximization explains human risk preference dynamics under changing environment
Source: PLoS Comput Biol. 2024 May 13;20(5):e1012080. doi: 10.1371/journal.pcbi.1012080 (PMC11115364; doi:10.1371/journal.pcbi.1012080)
Supplement: S4 Table — ***p < 0.001 (Bonferroni corrected). (PDF) [file pcbi.1012080.s004.pdf]

**Experiment 1**		EV	Risk	Δp_c	Δp_p	Prev. choice	Prev. reward	Prev. success	Acc. reward
Correlation	Risk	0.90***							
	Δp_c	0.24***	0.00						
	Δp_p	0.00	0.00	-0.54***					
	Prev. choice	-0.01	-0.00	-0.18***	0.18***				
	Prev. reward	0.00	0.00	-0.13***	0.12***	0.41***			
	Prev. success	0.00	0.00	-0.17***	0.16***	0.67***	0.83***		
	Acc. reward	-0.00	-0.00	0.07***	-0.10***	-0.04***	-0.01	-0.02	
VIF		9.21	8.45	2.52	1.75	2.14	3.70	5.64	1.01

**Experiment 2**		EV	Risk	Δp_c	Δp_p	Prev. choice	Prev. reward	Prev. success	Acc. reward
Correlation	Risk	0.90***							
	Δp_c	0.31***	0.00						
	Δp_p	0.00	0.00	-0.41***					
	Prev. choice	-0.01	-0.01	-0.07***	0.10***				
	Prev. reward	-0.01	-0.01	-0.05***	0.07***	0.37***			
	Prev. success	-0.01	-0.00	-0.07***	0.09***	0.64***	0.82***		
	Acc. reward	-0.01	-0.00	-0.00	-0.05***	-0.03***	0.01	0.00	
VIF		13.16	11.65	3.01	2.54	1.95	3.53	5.18	1.33
