# Supplementary figures and images for: Foraging in a non-foraging task: Fitness maximization explains human risk preference dynamics under changing environment
Source: PLoS Comput Biol. 2024 May 13;20(5):e1012080. doi: 10.1371/journal.pcbi.1012080 (PMC11115364; doi:10.1371/journal.pcbi.1012080)

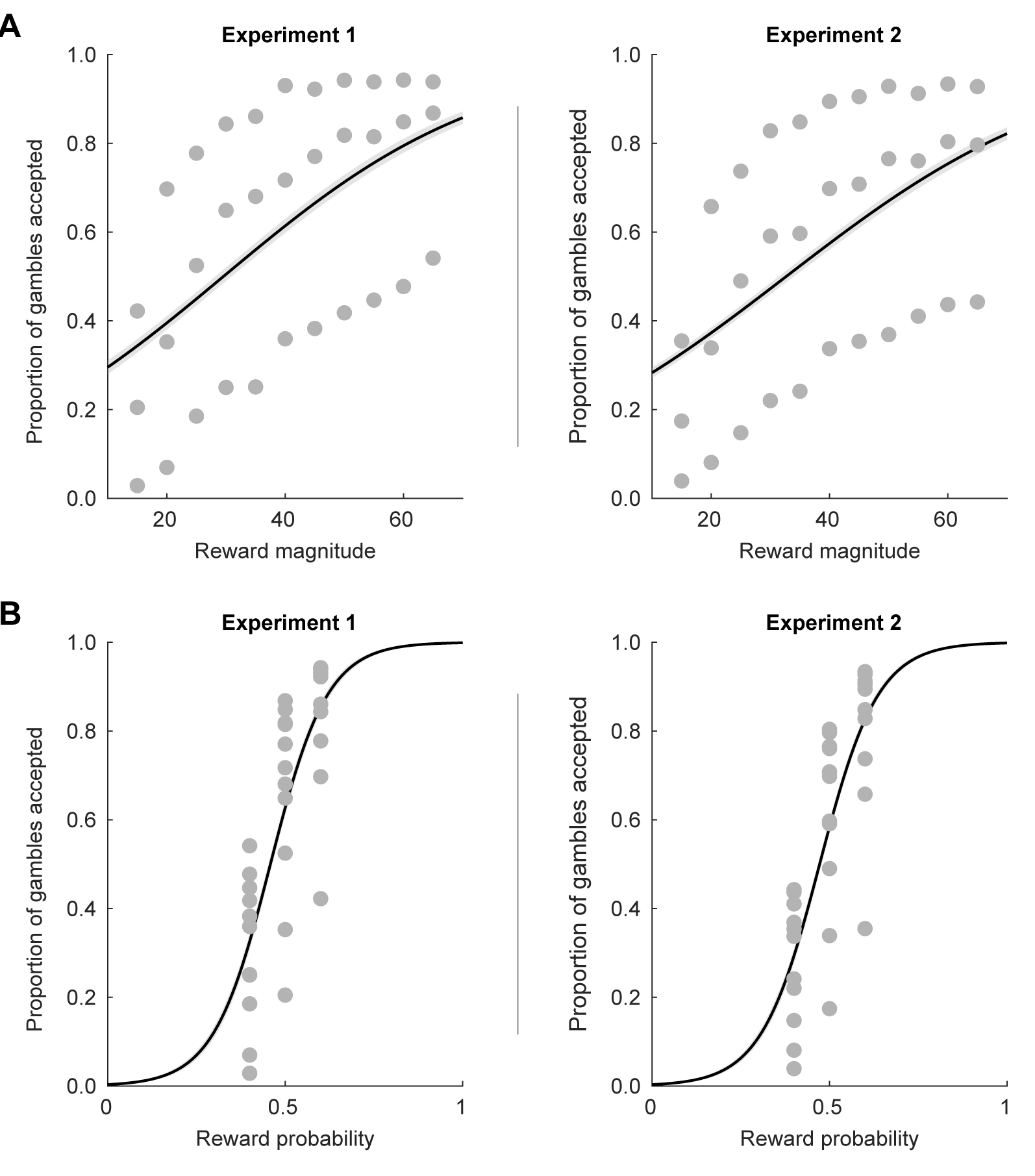

Supplement: S1 Fig — Proportions of gambles accepted for various combinations of gambling parameters were computed across all participants in each experiment, and sigmoid curves were fitted using binomial regression. (A) Binomial regression results with reward magnitude as the explanatory variable showed a significantly positive slope for the sigmoid curve in both Experiment 1 (Left, slope = 0.044, 95% CI = [0.040, 0.049]) and Experiment 2 (Right, slope = 0.041, bootstrapped 95% CI = [0.038, 0.045]). (B) Similarly, binomial regression results with reward probability as the explanatory variable demonstrated a significantly positive slope for the sigmoid curve in both Experiment 1 (Left, slope = 12.5, bootstrapped 95% CI = [11.4, 13.6]) and Experiment 2 (Right, slope = 12.1, 95% CI = [11.3, 13.1]). (TIF) [file pcbi.1012080.s005.tif]

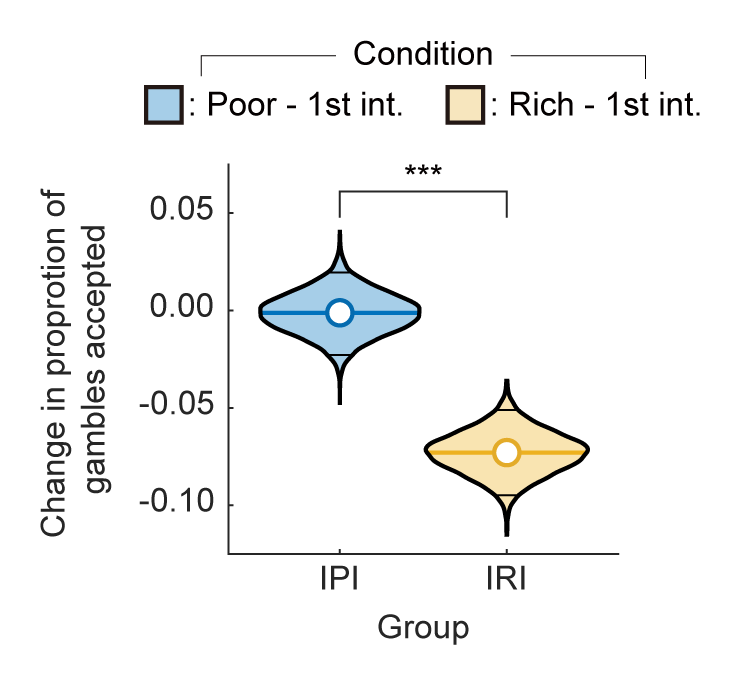

Supplement: S2 Fig — A significance test for the effect of environmental richness on gambling propensity. Change in the proportion of gambles accepted from the first to the second environment are computed for each group and compared (bootstrap test, one-tailed). Note that in the first environment both groups experienced the intermediate environment, and in the second block IPI and IRI group experienced the poor and rich environments, respectively. The shaded regions represent the variation, due to random samplings of participants, in the mean change in the proportion of gambles accepted as computed by bootstrap. The colored and black horizontal lines correspond to the mean and 95% confidence intervals, respectively. ***p < 0.001. (TIF) [file pcbi.1012080.s006.tif]

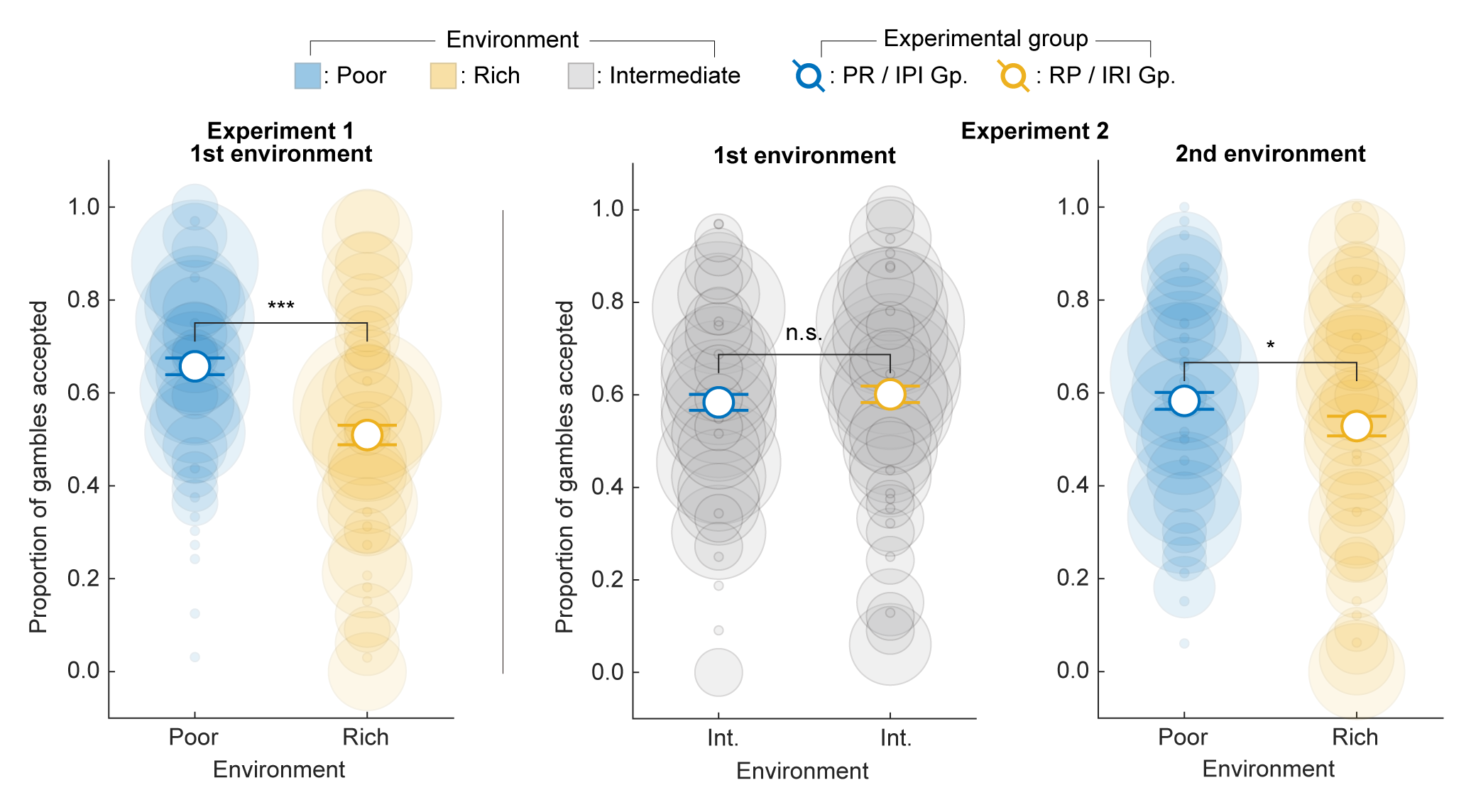

Supplement: S3 Fig — Mean proportion of gambles accepted by each group during the first environment of Experiment 1 (left) and during the first and second environments of Experiment 2 (right). In both Experiment 1 and Experiment 2 (second environment), participants accepted significantly more gambles during the poor environment than during the rich environment. In contrast, when both groups (IPI and IRI) were experiencing the same intermediate environment in Experiment 2 (first environment), the difference in the proportion of gambles accepted was not significant. One-tale bootstrap tests were used for the test of significance. The circles and error bars represent the mean and standard error. The bubble plots represent the distribution of the proportion of gambles accepted by each participant. *p < 0.05; ***p < 0.001. (TIF) [file pcbi.1012080.s007.tif]

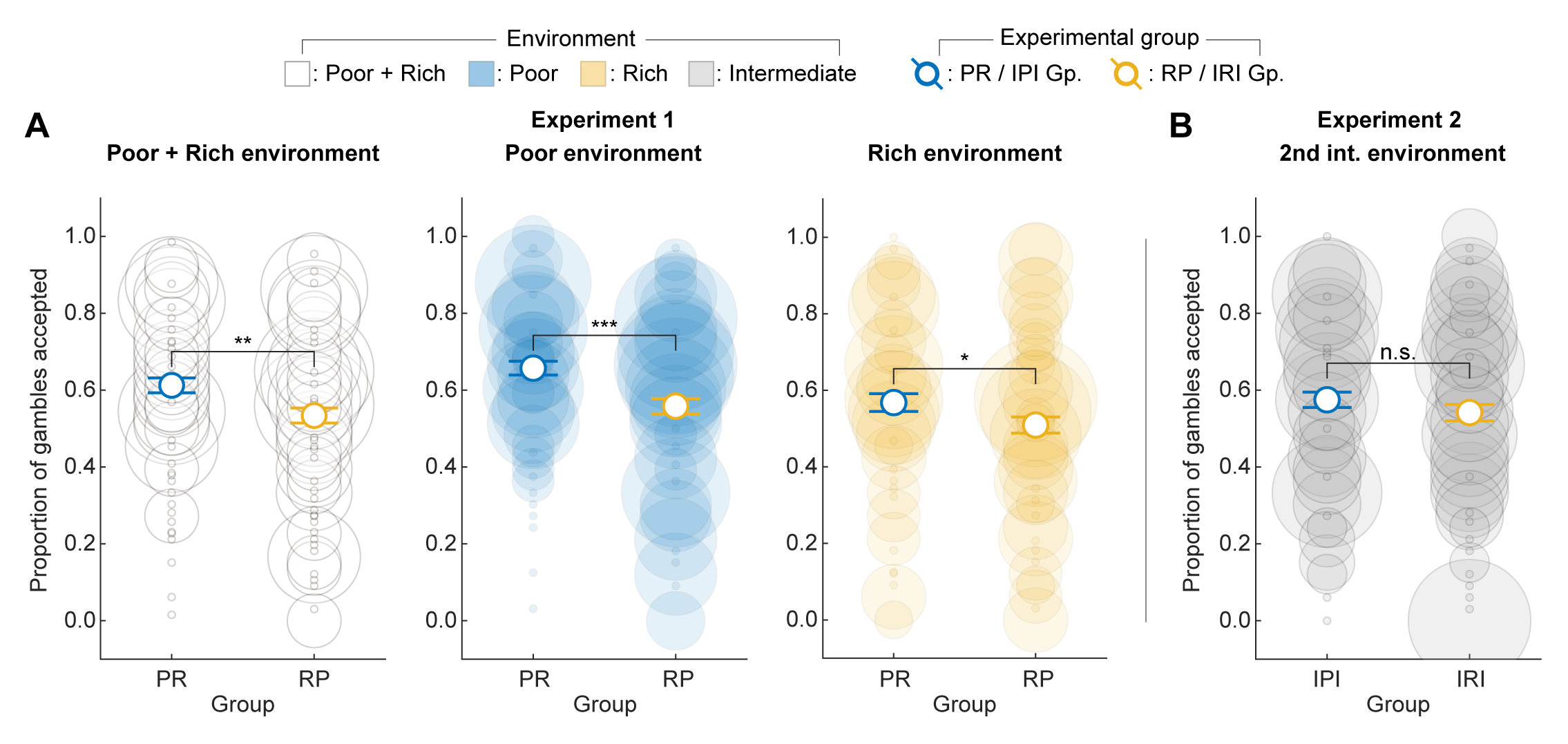

Supplement: S4 Fig — (A) In Experiment 1, the group that experienced the poor environment first (PR group) accepted significantly more gambles than the group that experienced the rich environment first (RP group) across the whole experiment (left), and in both the poor (middle) and rich (right) environments. (B) In Experiment 2, the IPI group accepted more gambles than the IRI group in the final block (second intermediate environment), although this trend did not reach significance. One-tale bootstrap tests were used for the test of significance. The circles and error bars represent the mean and standard error. The bubble plots represent the distribution of the proportion of gambles accepted by each participant. *p < 0.05; **p < 0.01; ***p < 0.001. (TIF) [file pcbi.1012080.s008.tif]

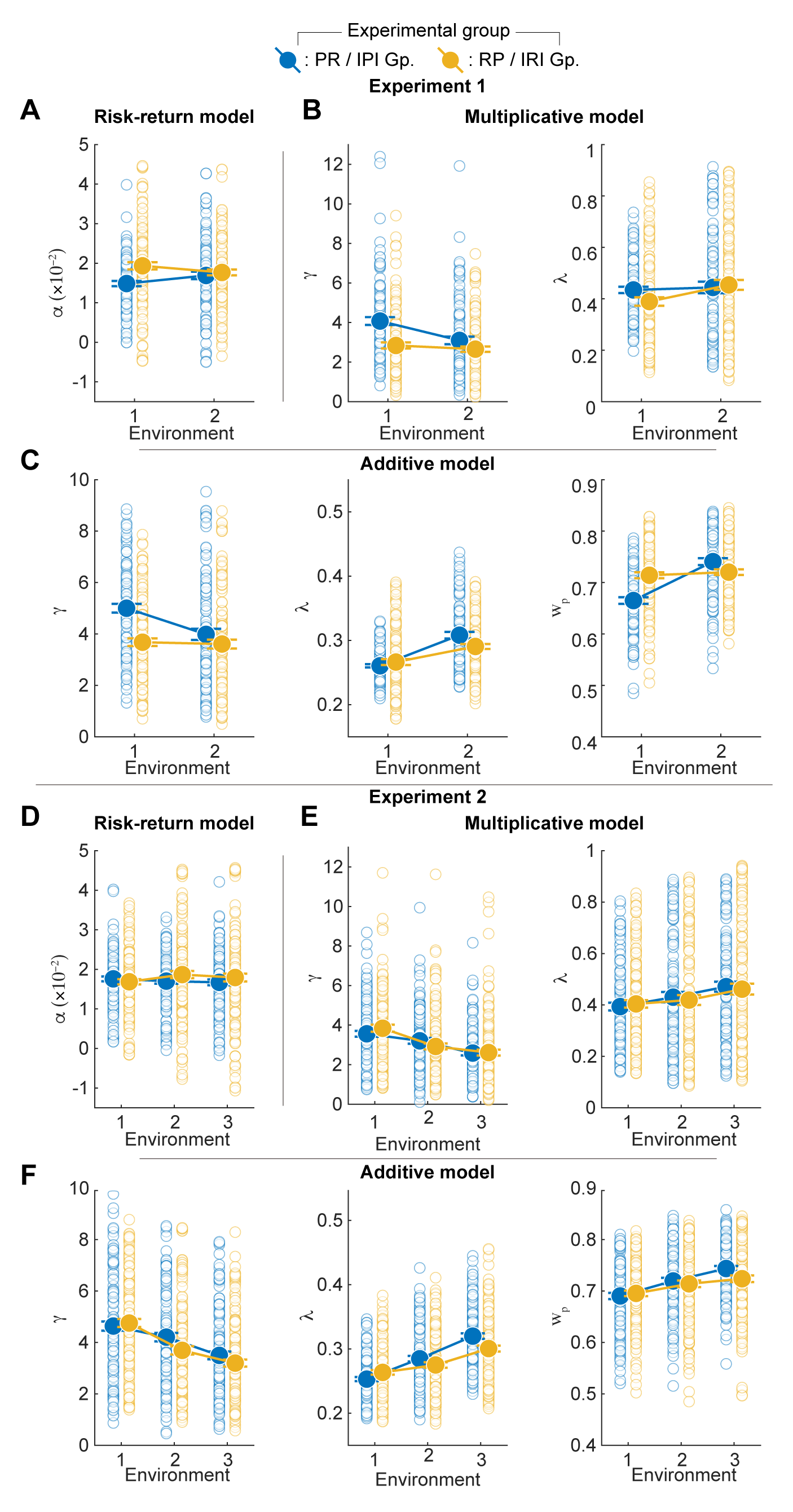

Supplement: S5 Fig — Model parameters were estimated from the trial-by-trial choices of each participant for each environment. (A) Risk-aversiveness α of the risk return model estimated for Experiment 1. (B) Risk-proneness γ of probability weighting function (left) and risk-proneness λ of the value function (right) of the multiplicative model estimated for Experiment 1. (C) Risk-proneness γ of probability weighting function (left), risk-proneness λ of the value function (middle), and risk-aversiveness wp (right) of the additive model estimated for Experiment 1. (D-F) Mirroring the format of A-C but are plotted for Experiment 2. The filled circles and colored bars denote the means and their respective standard errors. Unfilled circles signify the estimated values of the parameters for each participant within each environment. (TIF) [file pcbi.1012080.s009.tif]

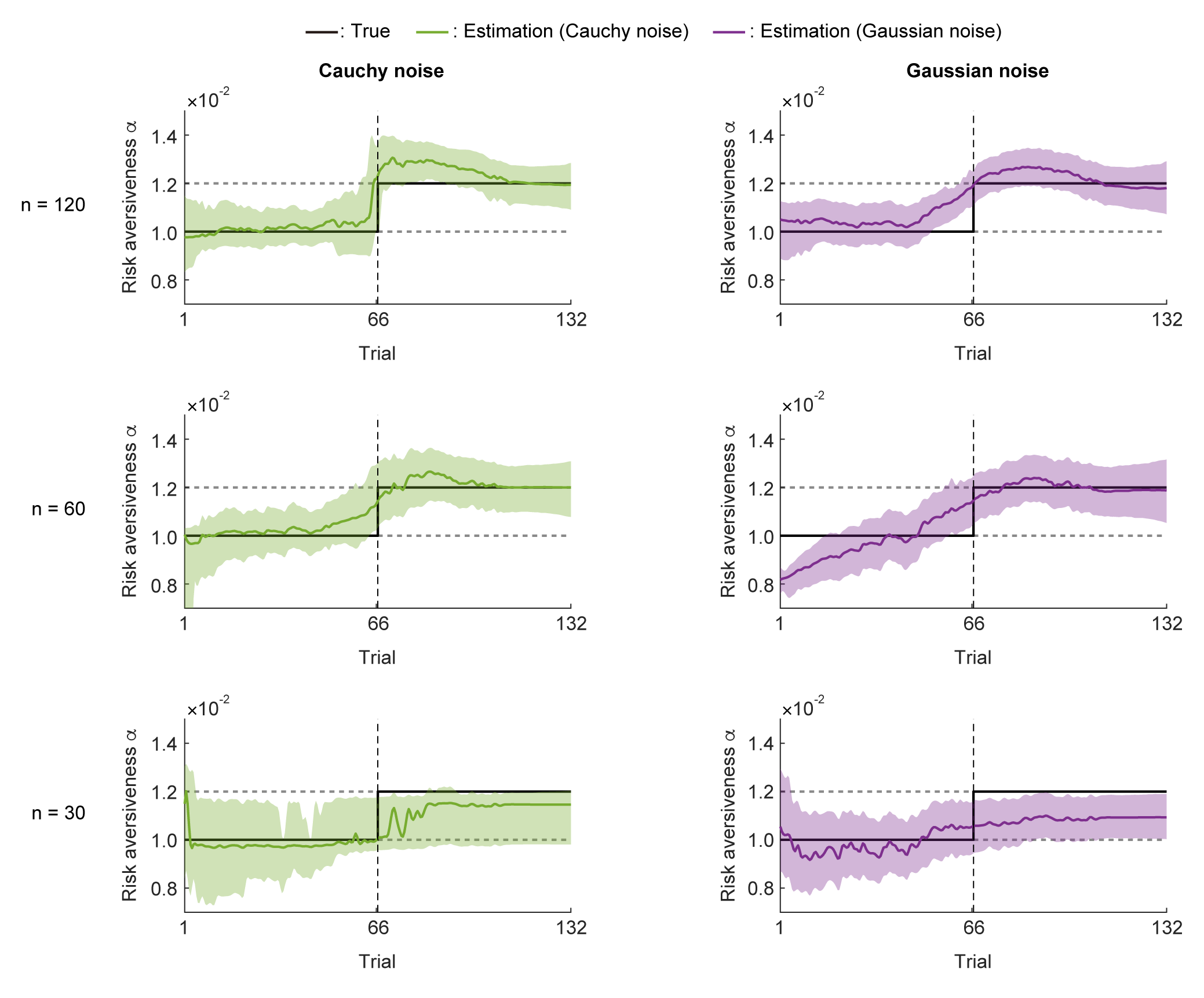

Supplement: S6 Fig — Choices were simulated assuming a step change in the risk-aversiveness α (black). Then, the particle filter was used to estimate the risk-aversiveness from the simulated choices (green and purple lines). The particle filter was able to recover the sudden change in risk-aversiveness when simulation parameters were set close to that of the real experiment (number of trials in a block = 66, number of participants in a group = 120, and Δα = 0.2×10^−2^) and Cauchy noise was assumed in the system model (green line, top left). The shaded regions represent the 90% credible intervals of the risk-aversiveness estimate by the particle filter. (TIF) [file pcbi.1012080.s010.tif]

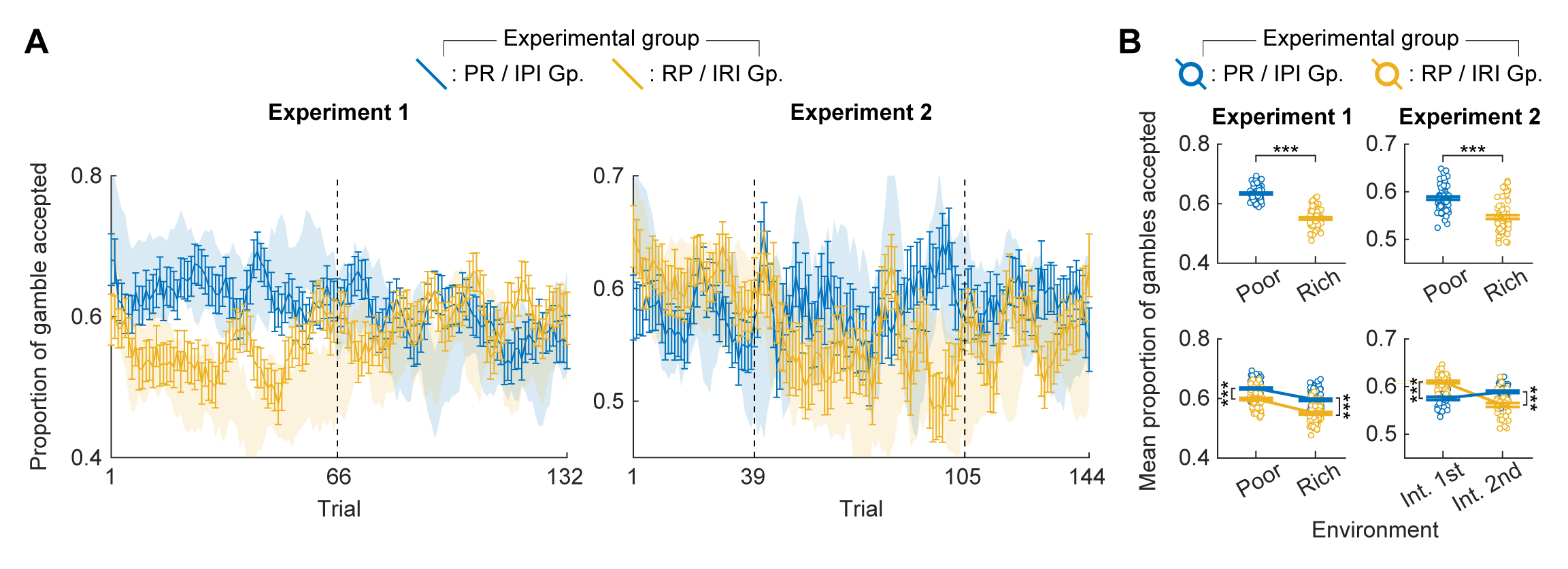

Supplement: S7 Fig — (A) A moving average of the proportion of gambles accepted is computed for the simulated choices of each group (n = 120) across Experiment 1 (left) and Experiment 2 (right). Each data point represents the proportion of gambles accepted in current trial and the preceding and succeeding two trials, forming a window size of 5 trials. Error bars represent standard errors. (B) Mean proportion of gambles accepted in each environment by each group. In both experiments, simulation showed that the proportion of the gambles accepted is significantly higher during the rich as compared to the poor environment (p < 0.001 for Experiment 1 and 2; top). In addition, the proportion of the gambles accepted was significantly higher after experiencing the poor rather than the rich environment (p < 0.001 for poor & rich environment in Experiment 1 and for the second intermediate environment in Experiment 2; bottom). One-tailed unpaired t-tests with Bonferroni correction were used for statistical comparisons of proportion of gambles accepted (except for the comparison for the first intermediate in Experiment 2 where two-tailed test was employed given the initial prediction that there is no difference.) ***p < 0.001. (TIF) [file pcbi.1012080.s011.tif]

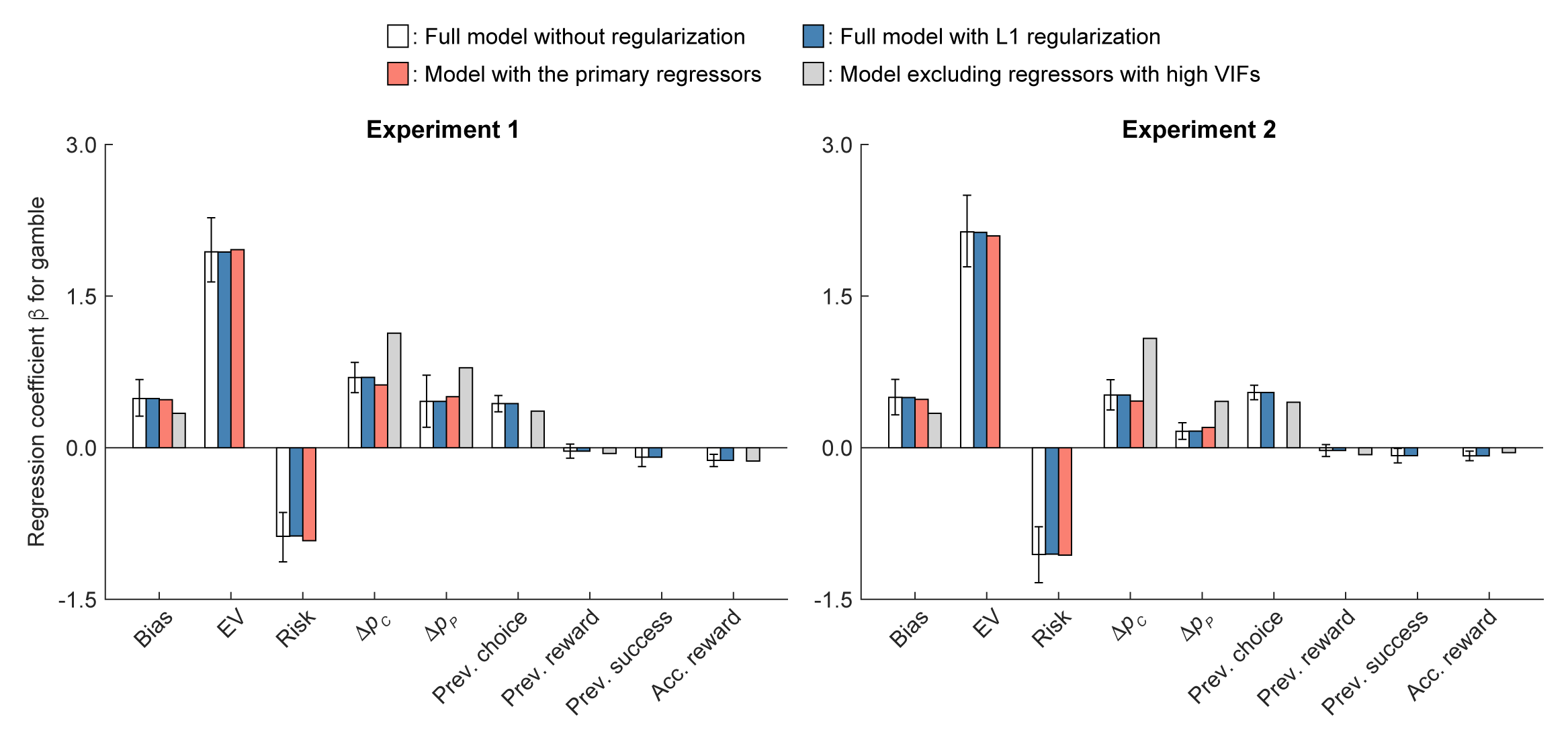

Supplement: S8 Fig — Regression coefficients estimated for each experiment with the full model (white; identical to Fig 5), full model with L1 regularization (blue), the model with only the primary regressors (EV, Risk, Δpc, and Δpp; pink), and the model excluding regressors with a VIF greater than five in either experiment (grey; see S4 Table for the values of VIF). Error bars represent 95% confidence bounds computed from bootstrap. (TIF) [file pcbi.1012080.s012.tif]

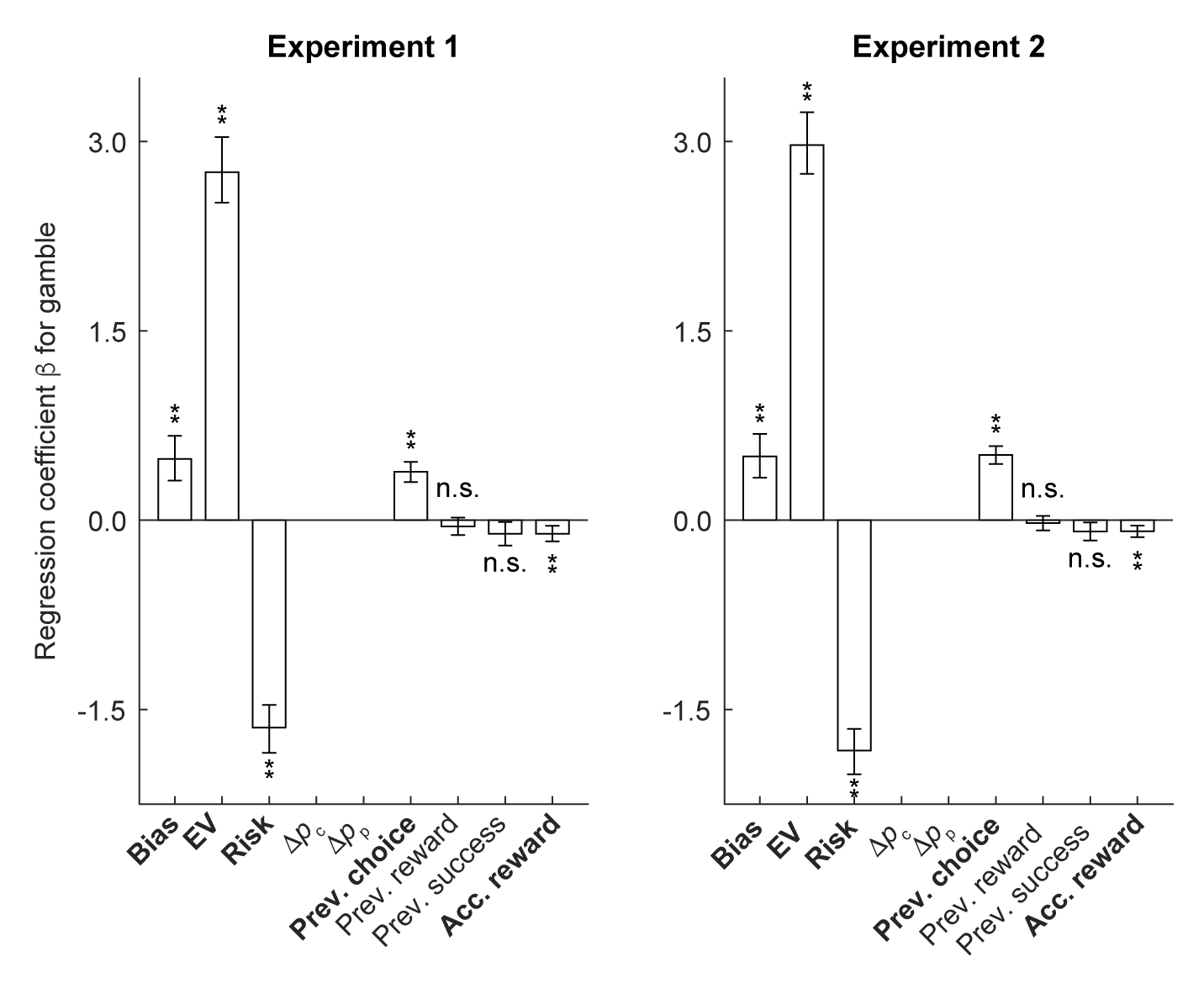

Supplement: S9 Fig — Mirroring the format of Fig 5, but plotted for the model excluding the two environment-related regressors, Δpc and Δpp. Regressors with consistently significant regression coefficients across the two experiments are noted in bold. Error bars represent 95% confidence bounds computed from bootstrap. Two-tailed one-sample bootstrap tests against zero with Bonferroni correction were used for statistical tests of regression coefficients. **p < 0.01. (TIF) [file pcbi.1012080.s013.tif]

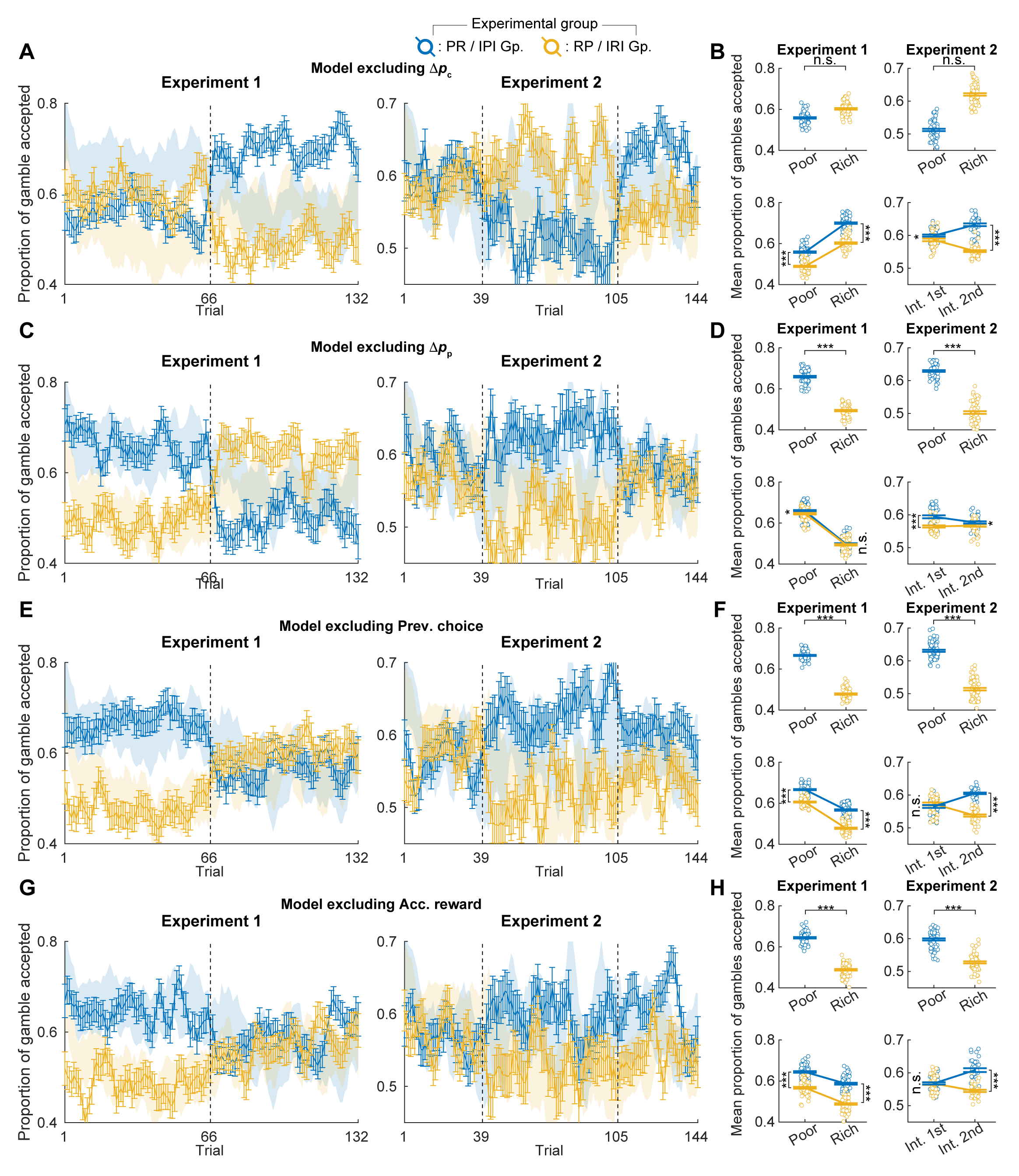

Supplement: S10 Fig — (A, B) The simulation results of the model excluding Δpc (n = 120 for each group). The moving average of the proportion of gambles accepted for each trial (A) and their environment-wise average (B) were calculated for each group. (C, D) Mirroring the format of A & B but plotted for the model excluding Δpp. (E, F) Mirroring the format of A & B but plotted for the model excluding Prev. choice. (G, H) Mirroring the format of A & B but plotted for the model excluding Acc. reward. (A, C, E, G) Error bars represent standard errors. Shaded regions are 95% confidence intervals of the proportion of gambles accepted computed for the real data. (B, D, F, H) Horizontal bars and unfilled dots represent the mean and raw values of the proportion of gambles accepted, respectively. One-tailed unpaired t-tests with Bonferroni correction were used for statistical comparisons of the mean proportion of gambles accepted (except for the comparison for the first intermediate in Experiment 2 where two-tailed test was employed given the initial prediction that there is no difference.) *p < 0.05; ***p < 0.001. (TIF) [file pcbi.1012080.s014.tif]

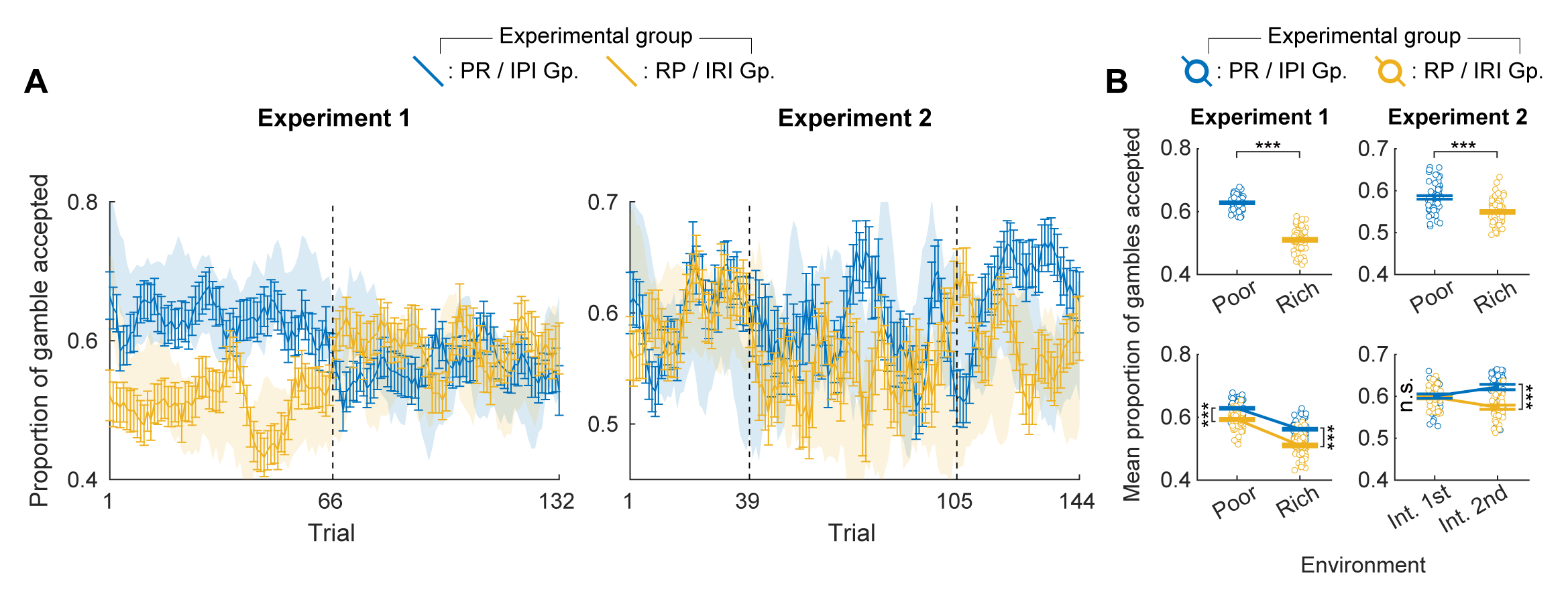

Supplement: S11 Fig — The simulation results of the model including regressors for two and three trials ago on top of the regressors in the original model (n = 120 for each group). (A) The moving average of the proportion of gambles accepted for each trial. Error bars represent standard errors. Shaded regions are 95% confidence intervals of the proportion of gambles accepted computed for the real data. (B) The mean proportion of the gamble accepted for each environment. Horizontal bars and unfilled dots represent the mean and raw values of the proportion of gambles accepted, respectively. One-tailed unpaired t-tests with Bonferroni correction were used for statistical comparisons of the mean proportion of gambles accepted (except for the comparison for the first intermediate in Experiment 2 where two-tailed test was employed given the initial prediction that there is no difference.) ***p < 0.001. (TIF) [file pcbi.1012080.s015.tif]
