# Supplementary material for: Foraging in a non-foraging task: Fitness maximization explains human risk preference dynamics under changing environment
Source: PLoS Comput Biol. 2024 May 13;20(5):e1012080. doi: 10.1371/journal.pcbi.1012080 (PMC11115364; doi:10.1371/journal.pcbi.1012080)
Supplement: Attachment — Submitted filename: Review PLOS Computational Biology - 2023.pdf [file pcbi.1012080.s016.pdf]

Review for "Foraging in a non-foraging task: fitness maximization explains human risk preference dynamics under changing environment"

This manuscript by Mochizuki et al. aims to investigate the role of fitness maximization in explaining variations in human risk preferences under changing environmental conditions, drawing parallels with foraging behavior in animals. The authors conducted an online task in which participants made decisions to accept or reject a risky gamble in two environments with varying average probability of reward (referred to as richness). The behavioral results indicate that human risk preferences vary over time and are influenced by both the richness of the environment and the order in which participants experience different environments. Logistic regression analysis was further employed to demonstrate that the relative attractiveness of a gambling option compared to other options in the current and past environments plays a significant role in explaining participants' propensity to gamble.

Overall, this manuscript effectively tackles a timely topic and is commendable for its clear and accessible presentation. The figures provided are informative and easy to interpret. However, there are a few major concerns that should be addressed to strengthen the manuscript.

First, the authors claim to demonstrate that "humans make risk choices to maximize long-term fitness rather than utility" (l.122-123). However, based on my understanding of the manuscript, the data presented does not fully support this conclusion. One major issue is the lack of a clear definition of the utility maximization and fitness maximization models that the authors seek to compare. It is essential to provide a precise explanation of these models and how they relate to the nested models used for the two simulations to draw reliable conclusions about their performance. The authors' conclusion relies on two simulated data sets: one including and the other excluding the environment-related regressors Δp_c and Δp_p. Nevertheless, the methodology employed in the second simulation (fig. 5B and 5D) is unclear. It appears that the simulation was conducted by excluding the two parameters of interest while using the same regression estimates as when those parameters were included in the regression, which is not an appropriate approach to address this question. To determine the necessity of including these two parameters in the model, it is essential to re-run the regression without them, compare the two nested models (e.g., using an F-test), and simulate the data based on the new regression estimates. Moreover, several variables used in the regression exhibit high correlation (e.g., Δp_c and EV, both of which incorporate the reward probability of the current option), resulting in multicollinearity. Addressing multicollinearity is crucial for accurately interpreting the effects of individual independent variables on participants' choices. To revise their modeling approach, the authors may find it beneficial to consult Wilson & Collins (2019) article on computational modeling of behavioral data.

Secondly, the regression analysis failed to demonstrate that accumulated reward predicted behavior in Experiment 2 (l.471-473). However, the effect of accumulated reward is an integral part of the authors' definition of risk-sensitive foraging theory (l.90-92). Consequently, without a clear definition of their model (as previously mentioned), it is premature to conclude that this theory explains the observed behavior in their experiment.

Finally, another significant concern is the lack of discussion on how these findings relate to previous research in this area. Although the subject matter appears to fall within the broader field of context-dependent preferences (see, for example, Palminteri & Lebreton, 2021), no related literature is cited in the manuscript, particularly concerning the existing models proposed to explain this phenomenon (e.g., divisive or range-normalization models). It is essential for the authors to revise their Introduction and Discussion sections to incorporate references to the relevant literature. They should elucidate the differences between their proposed model and existing models, as well as demonstrate how their new model offers a superior explanation of behavior in this task compared to previous models.

Minor points :

- It is unclear how many trials were conducted in each block. Clarification on this matter is needed:
  - In figure 1B, there are 60 trials in the poor and rich environments, and 36 trials in the intermediate environment (with reward magnitude varying from 10 to 65).
  - In line 586-590, the manuscript states that "in the poor, intermediate, and rich environments, reward probabilities were uniformly distributed with p = {0.2, 0.3, 0.4, 0.5, 0.6}, p = {0.4, 0.5, 0.6}, and p = {0.4, 0.5, 0.6, 0.7, 0.8}, respectively (Fig 1B, x-axis). The distribution of reward magnitude was the same for each reward probability and set as m = {15, 20, 25, …, 65} (Fig 1B, y-axis)."
    This implies that there were 55 trials per block in the poor and rich environments, and 33 trials per block in the intermediate environment.
  - l.597-599: "The total number of trials during the rich and poor environments was 66, and that during the intermediate environment was 39"
- The total number of catch trials in each block is not specified.
- Please provide information on the average number of failed trials due to slow response times.
- According to Figure 1B, the choices with a reward magnitude of 10 have been excluded from the analyses. However, this exclusion is not mentioned in the text. The rationale for removing these trials should also be explained.
- The text mentions that "two groups of participants experienced different sequences of environment settings" in each experiment (l.600-601), but the number of participants per group is not specified. It is important to clarify this information (e.g., whether it was half of the participants).
- The number of participants indicated in the method does not match the number of participants in the downloadable data files, both before and after applying exclusion criteria.
- Please report t-test results in the following format: Mean, Standard deviation, *t* (degrees of freedom) = *t*-value, *p* = *p*-value
- Can you provide statistical analyses supporting the following statements from the manuscript:
  - "The participants chose to gamble more frequently as the reward magnitude (x-axis of Fig 2A) and reward probability of the gamble (difference of the line types in Fig 2A) increased" (l.172-174)
  - "In addition, we noticed that the difference between the IPI and IRI groups during the second block of experiment 2 is smaller than the difference between the PR and RP

groups in the first block of experiment 1." (l.257-259). Please also include a reference to Figure 2B.
  - "For experiment 1, the particle filter estimates for risk aversiveness were on average higher for the RP group than for the PR group" (l.270-271)
- Figures 3C and 3D top right are not discussed in the text.
- Figures 3C and 3D right: the meaning of dots and horizontal lines is not mentioned in the legend.
- Please provide clarification on the formulas used to compute Δp_c and Δp_p. Were the formulas as follows?
  - $\Delta p_c =$ Reward probability of the current option – Maximum reward probability of the environment
  - $\Delta p_p =$ Maximum reward probability of the current environment – Maximum reward probability of the previous environment
- Can you specify how expected value (EV) and risk were computed?
- The link made between "the relative attractiveness of gambling options in the current environment compared to those in the past environments" and "an affect-based psychological mechanism" (l.474-478) is difficult to understand. Please provide clarification on this point.

Bibliography

Palminteri, S., & Lebreton, M. (2021). Context-dependent outcome encoding in human reinforcement learning. *Current Opinion in Behavioral Sciences*, *41*, 144-151. https://doi.org/10.1016/j.cobeha.2021.06.006

Wilson, R. C., & Collins, A. G. (2019). Ten simple rules for the computational modeling of behavioral data. *eLife*, *8*, e49547. https://doi.org/10.7554/eLife.49547
