# Supplementary material for: Foraging in a non-foraging task: Fitness maximization explains human risk preference dynamics under changing environment
Source: PLoS Comput Biol. 2024 May 13;20(5):e1012080. doi: 10.1371/journal.pcbi.1012080 (PMC11115364; doi:10.1371/journal.pcbi.1012080)
Supplement: Attachment — Submitted filename: Response20240404.pdf [file pcbi.1012080.s018.pdf]

Point-by-point response to the reviewers

Dear Prof. Stefano Palminteri and Prof. Daniele Marinazzo,

We are delighted to learn that our manuscript has been accepted with minor revisions. We sincerely thank you for editing our manuscript. We have now uploaded generating codes for the figures in the manuscript to the Github repository as referenced in L.1026 (https://github.com/yazmochi/forageNonforage).

We also extend our gratitude to the reviewers for their thoughtful feedback on our manuscript. Their comments and suggestions were invaluable, guiding us toward significant revisions and enhancements of our work. Detailed responses to the minor revision suggested by Reviewer #2 are provided below.

Reviewer #2: I appreciate the authors' efforts in addressing my previous concerns. I believe they have made satisfactory adjustments that have greatly improved the manuscript.

Thank you for your positive evaluation on the revision of our manuscript. We believe that the document has benefited significantly from your suggestions. We address your minor concerns point by point below.

However, I would like to provide some minor suggestions and noticed a few typos that could be corrected:

1. In Figure 1D, it seems to me that the circles represent the optimal risk preference, but this information is not mentioned in the legend. Adding this information would be helpful.

The information is now provided in L.197.

2. L.216: In this section, the authors mention "participants' needs" as the minimum number of points within a set of trials required for survival (Fig 1D). To enhance clarity, it would be helpful if the authors explicitly specify the number of points considered for high, moderate, and low needs.

The information is now provided in L.195-196:

> “In high, moderate, and low need conditions, the minimum number of points required for survival after 60 trials were set at 850, 1000, and 1150 points, respectively.”

3. L.377: The statement mentions, “The predicted changes in risk-preference parameters were significant for the risk-aversiveness of the risk-return model, and the risk-proneness of the

additive model, consistently across Experiment 1 and Experiment 2." However, based on my interpretation of S7 Table, the risk-proneness of the additive model is not consistently significant across both experiments. Clarification on this point is needed.

Thanks for spotting this mistake, we have removed the mentioning of the risk-proneness of the additive model (L.381).

4. L.389: "The model comparison of the approach-avoidance version of the models with iBIC showed that the best model was the additive model including both and (S8 Table)". It seems to me that there may be a reference error and it should be S5 Table instead.

   Thanks again for spotting this typo, we have now referenced S5 Table (L.393).

5. Model Choice in Particulate Filter: In the results section, it would be helpful if the authors explained why they continue to use a risk-return model in the particulate filter when the additive model consistently outperforms it in the previous section.

   We have now added a brief explanation for the rationale behind the use of a risk-return model (L. 1177-1180): "We adopted the risk-return model because it demonstrated superior performance among models featuring two parameters—one for risk preference and the other for choice stochasticity. This model provides simple and interpretable insights into the temporal dynamics of risk preference."

6. L.550: I believe there is a typo in the term "Acc. Reared" and it should be corrected to "Acc. Reward".

   Thanks again for spotting this typo, we have now corrected the spelling (L.554).

7. It would be helpful if the authors provided an explanation of the rationale for using iBIC for fitting computational models and BIC for regression analysis. Clarifying the differences between the two methods would be appreciated.

   We have now added a brief explanation for the rationale behind the use of iBIC (L. 1137-1140): Since we employed a hierarchical computational model with a group-level prior distribution, we used the integrated Bayesian Information Criterion (iBIC) for model selection, following the recommendation of the previous study [65]. Lower values of iBIC indicate a more favorable model fit.

8. L.639: The statement mentions, “Next, we simulated participants' choices with significant regressors common to both experiments (shown in bold in Fig 5).” However, it appears that Δ and Δ are not bold in Fig. 5 as indicated.

Thanks again for spotting this mistake, we have revised Fig 5.
