# Supplementary material for: Foraging in a non-foraging task: Fitness maximization explains human risk preference dynamics under changing environment
Source: PLoS Comput Biol. 2024 May 13;20(5):e1012080. doi: 10.1371/journal.pcbi.1012080 (PMC11115364; doi:10.1371/journal.pcbi.1012080)
Supplement: Attachment — Submitted filename: Response20240203.docx [file pcbi.1012080.s017.docx]

Point-by-point response to the reviewers

Dear Prof. Stefano Palminteri and Prof. Daniele Marinazzo,

We first apologize for the delay in revision of our manuscript and thank you for your understanding. The extra time greatly helped us in better addressing the reviewer’s comments.

We also thank the reviewers for their insightful comments on our manuscript. All the comments and suggestions were very helpful and guided our efforts to substantially revise and improve the manuscript. Our detailed responses are provided below.

Reviewer 1 ‧‧‧ page 2

Reviewer 2 ‧‧‧ page 17

Reviewer 3 ‧‧‧ page 31

**Reviewer #1**

This manuscript is about risky decision making about accept-reject reward gambles, combining it with an interesting concept from ecology, i.e. dynamic contextual changes in risk sensitivity (primarily described in the risk sensitive foraging literature, but more recently applied to human behaviour and neuroimaging). What is particularly novel about this study, compared to earlier work, is that it takes such an ecological view explicitly towards “non-foraging” type gambles, i.e. gambles without actual sequential dependencies or homeostatic needs. The authors show convincingly that regardless of this, something is clearly going on with participants risk taking, that cannot be explained simply through the notion of participants making independent trial by trial gambling decisions without contextual dynamics playing a part. Overall, this is a great idea, well executed and with convincing data. However, I think the manuscript could nonetheless benefit from some more clarifying analyses on the nature of their effects, some clarifications and some more contextualization in regards to the literature. Below a point-by-point breakdown of my comments:
Thank you for your positive evaluation of our manuscript. We believe that the document has benefited significantly from your suggestions. We address your concerns point by point below.

Major:
1) I very much liked their idea of bringing ecological theories into decision sciences. However, at times I wasn’t quite sure how everything fit together. Normally risk sensitive foraging theory is primarily about homeostasis and setpoints. Even in their example of starving animals its about meeting a minimal amount to ensure survival that pressures animals to take risks. However, in their study there are no equivalent analyses that would suggest a specific setpoint. Of course, such analysis would be difficult to conduct as those thresholds would have to be bootstrapped somehow from the data and might differ between people, but that difference in the concept behind their inspiration and their approach should be noted. However, it is probably true that if participants have a reasonably high threshold for necessary earnings that this would push them towards increased risk taking in poor environments (and that this effect could persist if they don’t completely update their expectations about future gambles) and this could be potentially shown in a simulation but so far the authors do not explicitly show this link, making their results less about homeostatic decision making and more about general contextual risk modulation. **Response:** We express our gratitude to the reviewer for providing valuable suggestions. We have now incorporated the outcome of the suggested simulation into Fig 1D top (l.191-199; provided below). In particular, we varied the richness of the environment and participants' needs (defined as the minimum number of points required to survive within a certain number of trials) and plotted curves illustrating risk preference against the probability of survival. We found that the optimal risk preference shifts towards risk-prone behavior as the environment deteriorates and participant needs increase.

Fig 1 (D) Simulation of participants’ choices. Participants’ choices were generated using the risk-return model (Method), and risk-aversiveness $\alpha$ is plotted against survival probability (top) and mean accumulated reward (bottom) for the poor (blue) and rich (orange) environments and varying need (represented by different lines). The optimal risk preference for survival probability varies with specific environmental and need conditions. As the environment worsens and needs increase, optimal risk-aversiveness shifts towards risk-prone behavior (decreases toward $\alpha<0$).

This is in accordance with the analysis of the participants’ real choices. The change in the simulated optimal risk preference in different environment and need conditions mirrors the effect of the current and past environments in the real choice data, respectively.

In addition, we commented on the challenge of directly elucidating the survival threshold equivalent of the points earned by participants in the discussion as follows (l.850-859):

“*Our current study has several limitations. First, while the fitness maximization framework correctly predicted the behavior of the participants in our experiments, a critical constraint is our inability to directly measure the participants' internal needs or thresholds which may drive their behavior. However, we note that in animal foraging, it is unlikely that animals adjust their behavior through a direct computation of energy requirements (need); rather, they likely rely on the feeling of hunger (affect). Similarly, we speculate that in our experiments, participants adjusted their choice propensity based on the subjective attractiveness of the option, rather than through a direct computation of need per se. This speculation is partly supported by the results of the regression analysis, but future studies are needed to further elucidate this aspect.*”

2) I wasn’t quite sure about the correlational structure of some of the parameters entered into the regression analysis. It would be very useful to see a correlation matrix of all multiple regression analysis. Specifically, I was wondering whether some regressors such as previous reward, but more importantly accumulated reward aren’t quite correlated with the environment type?
**Response:** We have now included the full correlation matrix of the regressors used in our regression analysis (S11 Table). $\Delta p_{p}$, which encodes the difference in mean reward probability between the current and past environments, essentially serves as an indicator variable for the environment. Although statistically significant, the correlations between $\Delta p_{p}$ and Prev. reward (Experiment 1: 0.12, Experiment 2: 0.07) and $\Delta p_{p}$ and Acc. reward (Experiment 1: -0.10, Experiment 2: -0.05) were found to be week.

(l.1514) **S11 Table. Correlation structure and VIF of regressors entered into the main regression analysis.** ****p* < 0.001 (Bonferroni corrected).

Nevertheless, it is acknowledged that some regressors displayed moderate to strong correlations. Despite the bootstrapped confidence intervals of the regression coefficients indicating a reasonable stability in their estimated values, we conducted several additional tests to explore the potential influence of multicollinearity. The results are presented in Supplementary Figure S12.

(l. 1517) **S12 Fig. Stability of regression coefficient.** Regression coefficients estimated for each experiment with the full model (white; identical to Fig. 5), full model with L1 regularization (blue), the model with only the primary regressors (EV, Risk, $\Delta p_{c}$, and $\Delta p_{p}$; pink), and the model excluding regressors with a VIF greater than five in either experiment (grey; see S11 Table for the values of VIF). Error bars represent 95% confidence bounds computed from bootstrap.

Specifically, we performed a rerun of the regression with L1 regularization, which robustly estimates regression coefficients even in the presence of multicollinearity. Additionally, we repeated the regression with only the primary regressors (excluding EV, Risk, $\Delta p_{c}$, and $\Delta p_{p}$) and ran it again, excluding regressors that exhibited a Variance Inflation Factor (VIF) greater than five in either experiment (regressors for EV, Risk, and Prev. success were excluded).

Overall, the outcomes of these analyses confirmed that the estimated values of regression coefficients remained reasonably stable.

The following text has been added to the results section (l. 608-630):

*“Several regressors in our analysis were correlated and had a high variance inflation factor (VIF; see S11 Table). Although the bootstrapped confidence intervals of the regression coefficients indicated a reasonable stability in their estimated values, we conducted several additional tests to investigate the potential influence of multicollinearity. Specifically, we reran the regression with L1 regularization, which robustly estimates regression coefficients in the presence of the multi-collinearity. In addition, we reran the regression with i) only the primary regressors (i.e., the regressors other than EV, Risk,* $\Delta p_{c}$*, and* $\Delta p_{p}$ *are excluded), and ii) excluding regressors that exhibited a VIF greater than five in either experiments (i.e., the regressors for EV, Risk, and Prev. success were excluded; S12 Fig). The estimated coefficients from both the L1 regularized regression and the regression with only the primary regressors showed no significant differences compared to those estimated from the original regression. (one-sample bootstrap test against the estimated value in the original model, two-tailed with Bonferroni correction, all p > 0.05). Significant differences were observed only in the regression that excluded regressors with high VIFs. Specifically, the regression coefficient for* $\Delta p_{c}$ *was significantly different for both Experiment 1 and Experiment 2 (both p = 0.001), and that for* $\Delta p_{p}$ *was significantly different in Experiment 2 (p = 0.001). The elevated values of these regression coefficients are likely due to the removal of the EV regressor, which exerted the largest influence on participants' choices and showed positive association with* $\Delta p_{c}$ *and* $\Delta p_{p}$*. Nevertheless, the estimated values of the coefficients consistently exhibited the same signs in all regression analyses, and the overall results confirm a reasonable stability of the regression coefficients estimated from our original model.”*

I am somewhat worried that the authors inadvertently omitted the nonmatched trials outcomes when computing the accumulated rewards, removing such correlations but distorting the meaning of accumulated rewards as it now does not reflect the actual reward experience of the participants anymore.
**Response:** We incorporated all trials when constructing the regressors. However, during the fitting of the regression model to the choices, we excluded trials other than the ones under analysis. As a result, Acc. reward is coded as the cumulative sum of rewards obtained in all past trials up to the previous trial. This clarification is now explicitly stated in Method (l. 1222-1223):

*“All regressors were computed for every trial, pooled for each experiment, and subsequently z-scored. The model fitting process was then carried out exclusively with the analyzed trials depicted in Fig 1B.”*

Additionally, related to point 1, accumulated reward related effects could be dynamic and based on where the agent is relative to their goals. In other words, testing the linear impact of accumulated reward might not be the right thing to do, but instead a binning of risk aversion or decision parameters by accum R bins to see whether there is a “peak”?
**Response**: We noticed that we had coded $\Delta p_{c}$ and $\Delta p_{p}$ regressors relative to the maximum reward probability in the current/past environment, which is not consistent with our hypothesis. We thus reanalyzed the data by coding the $\Delta p_{c}$ and $\Delta p_{p}$ regressors relative to the mean reward probability. **In this new regression model, the effect of accumulated reward was consistently significant in both experiments (Fig. 5).**

(l. 573-585) **Fig 5. Regressors capturing the relative attractiveness of a gambling option compared to other options in the current and past environments play a significant role in explaining participants’ gambling propensity.** Participants’ gambling propensity was positively correlated with the deviation of reward probability of the current gambling option from the mean reward probability of all gambling options in the current environment ($\Delta p_{c}$). Participants’ gambling propensity was also positively correlated with the difference between the mean reward probability in the current and past environments ($\Delta p_{p}$). Additionally, participants’ gambling propensity was negatively correlated with the accumulated rearward (Acc. reward). Regressors with consistently significant regression coefficients across the two experiments are noted in bold. Error bars represent 95% confidence bounds computed from bootstrap. Two-tailed one-sample bootstrap tests against zero with Bonferroni correction were used for statistical tests of regression coefficients. **p* < 0.05; ***p* < 0.01.

3) I couldn’t tell from the analysis, whether participants were generally risk averse or risk seeking and whether they came closer to the ideal risk proneness in the rich or poor environment as defined by optimal i.e. nonbiased decision making?
**Response:** Overall, participants are risk-averse both during the rich and poor environment. This choice tendency is explicitly noted in Result (l. 368-372):

*“Overall, the mean estimated value of* $\lambda$ *in the additive and multiplicative models were below one, and the mean estimated value of* $\alpha$ *in the risk-return model was above one for all environments across two experiments, indicating participants’ inclination to risk-averse choice tendencies (S6 Fig).”*

Additionally, I couldn’t find any comparisons of decision models that take different distortions into account such as KT-models or linear weighting of probability and magnitude rather than multiplication of the objective values. A linear weight analysis might be particularly interesting because it could test whether changes in context specifically impacts the use of magnitude or probability in risky decision making (on top of effects on their general risk taking bias).
**Response:** As response to your comment and other reviewers’ comments, we now included the results of prospect theory-based models, using both the multiplicative model ($U=v\left( m \right)\pi(p)$) as well as the additive model ($U=\left( 1-w_{p} \right)v\left( m \right)+w_{p}\pi(p)$). The results is presented in the newly added section “Fitting of computational models confirms the presence of the current and past environment effects (l. 331-405)” in Results and the definition of the models and the fitting method are detailed in “Computational models and their fitting (l.1086-1135)” in Methods.

The results did not give simple interpretation (l. 373-380):
*“We tested our predictions (one-tailed t-tests in the direction of our prediction with Bonferroni correction) regarding the changes in risk-preference for each model parameter of the best performing models separately (i.e.,* $\alpha$ *for the risk-return model with,* $\lambda$ *and* $\gamma$ *for the multiplicative model,* $\lambda$*,* $\gamma$ *and* $w_{p}$ *for the additive model; S7 Table). The predicted changes in risk-preference parameters were significant for the risk-aversiveness* $\alpha$ *of the risk-return model, and the risk-proneness* $\gamma$ *of the additive model, consistently across Experiment 1 and Experiment 2. This result underscores the influence of both the current and past environmental effects on participants' risk preferences.”*

This point is noted in Discussion as a weakness of this study (l. 876-885):
“*Third, related to the previous caveat, our results of model fitting (S5 Table, S6 Fig, S7 & 8 Tables) were inconclusive. The effects of the current and past environments were mediated by different risk parameters depending on the choice of the utility model (the risk-return model, and prospect theory-based multiplicative and additive models) and the choice rule (the sigmoid choice rule and the approach-avoidance choice rule). We also note that the prospect theory-based models might not be entirely suitable for the current experiments due to the constrained range of reward probabilities. Despite this, we hope that the robust observation of the current and past environment effects in our model-free analysis will prompt future studies to delve deeper into the computational underpinnings behind the observed modulations in risk preferences.*”

4) There is other literatures that could explain at least some of their findings. For example, RL has long had the idea of negative contrast effects, i.e. that learning happens in comparison to the current average reward rate. Generalized, such an effect could explain why participants accept more gambles in a poor environment, without resorting to foraging style language, as negative contrast effects can be beneficial in learning to identify good items in bad environments. Also, If people assume they lose time or have other opportunity cost for every acceptance, they might rationally reduce acceptance in rich environments compared to poor ones as they might implicitly believe that could “squeeze” one ore trial in in rich environments if they move on quicker (however, some of this reasoning is of course related to patch leaving theories).These concepts need to be discussed!
**Response:** Thank you for this suggestion. We have now discussed the foregoing issues as follows (l. 860-875):
*“Second, participants were not informed about the environment that they will experience. Thus, they had to learn the reward statistics of the environment to adjust their choice behavior, indicating that participants have robust reward memories. However, we did not assess such learning effects as we refrained from constructing and testing specific computational models with intricate utility functions tailored to specific contexts aimed at providing a mechanistic account for the observed shift in risk preference during our experiment. As such, we did not explicitly test models incorporating a dynamic utility function. Noteworthy concepts in reinforcement learning and decision-making literature, such as the negative contrast effect [60], reference-point centering [26,31,32], range adaptation [26,33,32], and divisive normalization* [1–6]*[24,27,29,30], are likely to provide mechanistic accounts to our findings. For example, the two environment-related regressors may be interpretable as range adaptions at two different time scales. The effects of environment-related regressors (*$\Delta p_{c}$ *and* $\Delta p_{p}$*) and their affect-based interpretation we offered shines light on the question why such adaptation occurs at the two different time scales. We hope that our findings lead to the exploration of computational models with adaptations at multiple time scales in explaining human economic behavior.”*

5) Lastly, I was a bit surprised that the effect when modelled as risk aversion or in a regression was so much larger compared to when calculated through propoertion of gambles accepted. In other words, why is the dynamic risk aversivness plus particle filter more powerful? This is not to question their results, I just lack intiuition on the source of this difference and likely the reader will as well.
**Response:** The model employed in the particle-filter analysis, specifically the state-space model with risk aversion as a latent/hidden variable, presupposes a temporal correlation in risk aversiveness. In contrast, in the analysis of proportion of gambles accepted, each choice is treated as an independent event. This disparity imparts additional statistical power to the estimation results obtained through the particle filter. We have now included a model-free time-resolved analysis of the proportion of gambles accepted, using windowed averaging across trials (Fig 4). In this analysis, the effects were comparable to those of the particle filter.

6) You stated “Participants received 50 JPY (approximately 0.4 USD) upon completion of the task. “This seems very low as payment or am I misunderstanding this and it isn’t the overall payment? Also were participants paid on points earned or not? This is important information for a gambling study (I apologies if it was mentioned somewhere but I missed it).
**Response:** The participants indeed received a flat payment of 50JPY for participation in the study and were not paid any bonus on the basis of points earned. This amount of compensation is comparable to other studies conducted on Lancers. For example, this study (Suzuki, M., & Yamamoto, Y., Proceedings of the 2022 ACM Conference on Information Technology for Social Good, 2022) compensated participants with 33 JPY, and this study (Lim, J. S., DiGRA Conference, 2019) compensated participants with 40 JPY.

This is noted in the Method as follows (l. 931-933):
*“Participants received a fixed amount of 50 JPY (approximately 0.5 USD at the time of the experiment) upon completing the task. This payment amount aligns with the compensation offered for similar tasks on Lancers.”*

Minor:
- Risk could be variance (highest at 50/50) or odds of not winning (highest at lowest probability) so specify earlier clearly which definition you are using (economists and psychologist use the word risk to mean different things and this can be confusing when not clearly stated which one is used).
**Response:** We thank the reviewer for pointing this source of confusion out and have clarified our definition of risk Introduction as follows (l.62-63):
“*In this context, “risk” specifically refers to the variance in the outcome of a choice option, as defined in economics.*”

- It is called “Risky choices” in english not “risk choices”
**Response:** Thank you very much for spotting these mistakes…
The manuscript has been corrected.

- Why did you not analyze the lowest mag (10) that overlapped between environments or is this a ploting mistake?
**Response:** The trials with a reward magnitude of 10 were designated as catch trials and, therefore, were excluded from the statistical analysis. In these trials, rejection resulted in the sure gain of 10 points, whereas acceptance led to the same gain only probabilistically. Consequently, if participants understood the task rules correctly, they should consistently reject in these trials. The information that the trials with a reward magnitude of 10 were catch trials has been added to the caption of Fig 1 as follows (l. 184-185):
*“Gambles with a reward magnitude of 10 were excluded from the analysis as they were catch trials (see Method for details).”*

- I didn’t understand why the simulation without the effect would show the opposite trend? Please clarify
**Response:** This phenomenon is attributed to the influence of Prev. choice. The positive regression coefficient for Prev. choice suggests that participants tend to replicate their previous choices, leading to a higher likelihood of accepting (or rejecting) a gamble following a prior acceptance (or rejection). In a rich environment, where the mean reward probability is on average higher than in a poor environment, participants exhibit a greater tendency to accept gambles. The impact of Prev. choice amplifies this inclination, resulting in the trend where participants become more risk-prone in the rich environment than in the poor environment. This shift in participants' risk propensity contrasts with the expected and observed effects associated with the current environment, and as the result in the simulation where the regressor for $\Delta p_{c}$ is excluded, we observe a reversed trend.

- It wasn’t always clear why bootstrapping analyses were used (I understand it for the particle filter but some rather conventional measures also had it without explanations e.g. “Choices shown in A were pooled for each environment and the proportion of gamble accepted were compared between environments (bootstrap test, one-tailed). The shaded regions represent the variation, due to random samplings of participants, in the mean of the proportion of gambles accepted as computed by bootstrap. The colored and black horizontal lines correspond to mean and 95% confidence intervals of the proportion of gambles accepted, respectively. *p < 0.05; ***p < 0.001.”
**Response:** The adoption of bootstrap hypothesis testing for the environment-wise analysis of the proportion of gambles accepted was necessitated by the nature of our approach. Given that we computed and compared the proportion of gambles accepted by each group within each environment, this analysis yielded a single numerical value for each group. Consequently, the use of a t-test for between-group comparison is not applicable. The bootstrap method provided a robust alternative for conducting hypothesis testing in this specific context. This point has now been explicitly noted in Method (l. 1059-1063):
*“As the proportion of gambles accepted by the* $i^{th}$ *group* $q_{i}$ *represents a single value characterizing the choice tendency of a specific group, t-test is not applicable for the between-group comparison. Consequently, to capture the variability in the proportion of gambles accepted (Figs 2B and 3) or other statistics computed for pooled data (Figs 2A and 5), we performed bootstrapping at the participant-level [62].*”

- Fig 2. Could be clearer as it took me a long time to understand what was being plotted. Maybe have separate legends for subpanels but make them clearer?
**Response:** Thanks for this suggestion. In response, we have updated the legends in Fig. 2, and we believe that these revisions have enhanced the clarity and ease of interpretation.

(l. 220) **Fig 2. Participants accepted significantly more gambles in the poor environment than in the rich environment.**

- It would be good for readers if additional explanation of the rationale behind Experiment 2 happened earlier and was more thorough.
**Response:** We included the explanation in the first paragraph of Results as follows (l. 165-169):
*“The rationale behind introducing Experiment 2 was to assess the consistency of our results across different experiments and to control for the influence of the environment that participants had encountered in previous trials. In both experiments, participants were not given instructions regarding the presence of distinct environments or the sequence in which they would encounter them.”*

- In Fig 3 A make y axis all the same so that the data points can be compared across subpanels.
**Response:** We thank the reviewer for this helpful suggestion. Fig. 3A in the revised manuscript been replotted so that all panels have the same range in y axis:

(l. 297) **Fig 3. Effects of the past environment on participants’ risk preference.**

- You stated “(B) In experiment 2, the IPI group accepted more gambles in the second intermediate environment than the IRI group, although this trend did not reach significance (bootstrap test, one-tailed). “ but if it isn’t significant I wouldn’t use this language just because one is numerically higher than the other, as people normally assume statistical significance if you say “x is higher than y”. you can say “while x was numerically higher than y, it was not statistically significantly so”
**Response:** Thank you. As suggested, Fig. 3 caption has been revised as follows (l. 301-304):
*“(B) In Experiment 2, while the IPI group accepted more gambles in the second intermediate environment than the IRI group, this trend did not reach significance (bootstrap test, one-tailed).”*

- I was surprised how big the previous choice effect was. It suggests to me that the stickiness does not end there, but t-2 and t-3 probably also have an impact (although unlikely this would change any other findings).
**Response:** To verify this point, we have conducted additional analysis including the regressors for choices made at t-2 and t-3 time points, and plotted the result in S15 Fig. As you expected, inclusion of these regressors did not change the result, that we observed the current and past environment effects consistently.

(l. 1547) **S15 Fig. Incorporating regressors for the choices made two and three trials ago did not alter the outcome.** The simulation results of the model including regressors for two and three trials ago on top pf the original model (*n* = 120 for each group). (**A**) The moving average of the proportion of gambles accepted for each trial. Error bars represent standard errors. Shaded regions are 95% confidence intervals of the proportion of gambles accepted computed for the real data. (**B**) The mean proportion of the gamble accepted for each environment. Horizontal bars and unfilled dots represent the mean and raw values of the proportion of gambles accepted, respectively. One-tailed unpaired t-tests with Bonferroni correction were used for statistical comparisons of the mean proportion of gambles accepted (except for the comparison for the first intermediate in Experiment 2 where two-tailed test was employed given the initial prediction that there is no difference.) ****p* < 0.001.

The results are reported as follows (l.723-731):
*“Additionally, considering the relatively high values of the regression coefficients for the Prev. choice regressor (Fig 5), we conducted a subsequent regression analysis. In this iteration, the model incorporated not only the original set of regressors but also included additional regressors for choices made at two and three trials ago, and the moving average of the proportion of gambles accepted across the experiments are computed for the choice generated (S15 Fig A). We confirmed qualitative consistency between the proportion of gambles accepted computed for the simulated choices and the real choices for both Experiment 1 and Experiment 2, again recovering the effects of the current environment (S15 Fig B, top) and past environments (S15 Fig B, top).”*

- Its Experiment, not Experient. Saw this typo a couple of times.
**Response:** Thank you very much for spotting this typo…The manuscript has been corrected.

- Why is the change in risk aversion parameter in Exp2 so sudden in the middle bit but more smooth in Exp 1 in the simulation in Fig 5?
**Response:** First, we have removed the concerned figure from the manuscript in response to another reviewer’s comment.

Second, considering the discontinuous changes in the environment in our experiment, we modeled the risk aversiveness at the *t*-th trial, denoted as $\alpha_{t}$, to stochastically transition from the previous trial following a Cauchy distribution:

$$\begin{aligned} \alpha_{t} \sim\mathrm{Cauchy}\left( \alpha_{t-1}, \sigma\right).\#\left( Eq.15 in the maunscript \right) \end{aligned}$$

In contrast to stochastic transitions based on the Gaussian distribution, which are smooth and preclude sudden jumps, the Cauchy distribution, characterized by heavy tails, allows for abrupt changes on rare occasions. Consequently, when significant changes in the transition of risk aversiveness are observed, the model accommodates these changes by either expanding the flexibility parameter σ or maintaining it small and attributing it to a sudden jump on rare occasions. The choice between these explanations is determined through likelihood-based optimization applied to each group in each experiment.

For two groups in Experiment 1 and IPI group in Experiment 2, participants’ choices were better explained by relatively larger values of $\sigma$, resulting in smooth transitions of risk-aversiveness. Conversely, for IRI group in Experiment 2, the choices were better explained by a relatively small value of $\sigma$ with a significant jump in the middle.

For the two groups in Experiment 1, as well as for the IPI group in Experiment 2, participants' choices were more effectively explained by relatively larger values of $\sigma$, leading to smooth transitions in risk aversiveness. In contrast, for the IRI group in Experiment 2, the choices were better explained by a relatively small value of $\sigma$, indicating a significant jump in the middle of the experiment. These notions can be confirmed by inspecting the time-course of the proportion of gambles accepted across the experiment, now plotted as Fig. 4A in the revised manuscript.

- Why sure gains of 10 rather than probability of losing a certain amount? Not that this is wrong, but it would be nice to see an argument for it in the paper.
**Response:** We mentioned the rationale and limitation behind use of only gain gambles in discussion (l.886-889):
*“Fourth, our experiment exclusively involved gain-only gambles, intentionally omitting the exploration of the impact of losses to avoid the added complexity associated with loss aversion. Future studies may consider introducing sure losses to assess their effects.”*

- Their order effect you show can only be obtained if the participants have a good reward memory, which they appear to have. Might be useful to point that out.
**Response:** We thank the reviewer for this suggestion. We have now commented about the participants’ reward memory in the discussion as follows (l. 860-875):
*“Second, participants were not informed about the environment that they will experience. Thus, they had to learn the reward statistics of the environment to adjust their choice behavior, indicating that participants have robust reward memories. However, we did not assess such learning effects as we refrained from constructing and testing specific computational models with intricate utility functions tailored to specific contexts aimed at providing a mechanistic account for the observed shift in risk preference during our experiment. As such, we did not explicitly test models incorporating a dynamic utility function. Noteworthy concepts in reinforcement learning and decision-making literature, such as the negative contrast effect [60], reference-point centering [26,31,32], range adaptation [26,33,32], and divisive normalization [24,27,29,30], are likely to provide mechanistic accounts to our findings. For example, the two environment-related regressors may be interpretable as range adaptions at two different time scales. The effects of environment-related regressors (*$\Delta p_{c}$ *and* $\Delta p_{p}$*) and their affect-based interpretation we offered shines light on the question why such adaptation occurs at the two different time scales. We hope that our findings lead to the exploration of computational models with adaptations at multiple time scales in explaining human economic behavior.”*

**Reviewer #2**

**Review for “Foraging in a non-foraging task: fitness maximization explains human risk preference dynamics under changing environment”**

This manuscript by Mochizuki et al. aims to investigate the role of fitness maximization in explaining variations in human risk preferences under changing environmental conditions, drawing parallels with foraging behavior in animals. The authors conducted an online task in which participants made decisions to accept or reject a risky gamble in two environments with varying average probability of reward (referred to as richness). The behavioral results indicate that human risk preferences vary over time and are influenced by both the richness of the environment and the order in which participants experience different environments. Logistic regression analysis was further employed to demonstrate that the relative attractiveness of a gambling option compared to other options in the current and past environments plays a significant role in explaining participants' propensity to gamble.

Overall, this manuscript effectively tackles a timely topic and is commendable for its clear and accessible presentation. The figures provided are informative and easy to interpret. However, there are a few major concerns that should be addressed to strengthen the manuscript.
We want to convey our appreciation for your meticulous review of our manuscript and for sharing insightful comments. In the following sections, we methodically respond to each concern you brought.

First, the authors claim to demonstrate that “humans make risk choices to maximize long-term fitness rather than utility” (l.122-123). However, based on my understanding of the manuscript, the data presented does not fully support this conclusion. One major issue is the lack of a clear definition of the utility maximization and fitness maximization models that the authors seek to compare. It is essential to provide a precise explanation of these models and how they relate to the nested models used for the two simulations to draw reliable conclusions about their performance.
**Response:** We understand the concern regarding the lack of a clear definition of utility maximization and fitness maximization models. However, our intent was not to propose specific computational models to give a mechanistic account of participants behavior. Rather, we focused on unraveling the reason behind the employment of such mechanisms. Thus, our approach was deliberately descriptive, refraining from delving into detailed computational mechanisms for the utility functions. This principle extended to our regression analysis, where we included variables that might influence participants' choices but focused solely on their simple linear combinations.

We have revised our manuscript to clarify our perspective in Introduction and Discussion:

(Introduction, l. 69-81) *“However, the framing of the decision problem [8,9], the history of prior outcomes [10,11], an individual's emotional state [12–16], hormone levels [17–19], observation of other’s choices [20], and aging [21] can alter the risk preference of a decision-maker. The foregoing observations show that the constancy of risk preference does not always hold [22]. Numerous studies have endeavored to elucidate the mechanistic underpinnings of changes in risk preference by employing diverse computational models with intricate utility functions tailored to specific contexts [23–28]. Notably, models with divisive normalization [24,27,29,30] , reference-point centering [26,31,32], and range adaptation [26,33,32] have prominently featured in this exploration. However, while these studies proficiently elucidate the mechanisms through which dynamic modulation of risk preference arises as a consequence of learning-based utility modulation, they lack a comprehensive articulation of the adaptive significance that underlies the specific structure of the utility function and its evolution over time.*

*In contrast to these studies, ethological theories assume that animals’ risky choices are guided by the maximization of long-term fitness rather than utility, which naturally leads to dynamic risk preference [34,35]*.*.…”*

(Discussion, l.803-820) “*Behavioral economics and recent decision making literature investigate how human decision biases occur by developing computational models that explain observed behavior better than classical theories [6,48–50,23,26,24,30,27]. However, there is no consensus regarding why these biases occur in human economic decision-making [1,51]. To tackle this issue, our primary focus in data analysis centered on descriptive statistics (see Figs 2, 3, and 4A & B) rather than constructing and testing specific computational models aimed at providing a mechanistic account for the observed shift in risk preference during our experiment. We complemented this approach with a computational model with minimal assumptions. Specifically, we employed the risk-return model to encapsulate participants' risk aversion with a single model parameter within the utility function, and estimated its dynamic transition using a particle filter, which relies solely on the assumption of the Markov property in the temporal transition of the risk aversion parameter (see Figs 4C & D). This analytical strategy was deliberately chosen to contrast with the conventional method of analysis, wherein computational models are intricately detailed for the construction of utility functions and its dynamics [23–27]. In doing so, we aimed to provide a unique perspective by adopting an approach which allows us to test the qualitative predictions made by the frameworks of fitness and utility maximization, incorporating minimal assumptions in our computational accounts.”*

The authors' conclusion relies on two simulated data sets: one including and the other excluding the environment-related regressors ∆𝑝𝑐 and ∆𝑝𝑝.
**Response:** We have reframed the manuscript to clarify that our main results are the current and past environment dependent modulations of participants’ risk preference using model-free analysis of the proportion of gambles accepted. The regression analysis is introduced to support and strengthen our argument, and our overall conclusion does not hinge solely on its outcomes.

However, we acknowledge the methodological concern raised in your comments and those of Reviewer 3 regarding our original regression analysis. We appreciate your guidance, and based on your advice, we have thoroughly revised the manuscript. The specific modifications and improvements are explained in detail below.

Nevertheless, the methodology employed in the second simulation (fig. 5B and 5D) is unclear. It appears that the simulation was conducted by excluding the two parameters of interest while using the same regression estimates as when those parameters were included in the regression, which is not an appropriate approach to address this question. To determine the necessity of including these two parameters in the model, it is essential to re-run the regression without them, compare the two nested models (e.g., using an F-test), and simulate the data based on the new regression estimates.
**Response:** We thank the reviewer for pointing out this mistake on our part. We have now re-run the regression without them and used the new regression estimates for the second simulation (S13 Fig).

In addition, as suggested, we have incorporated the results of model comparison using Bayesian Information Criterion (BIC). (The choice of BIC over Akaike's Information Criterion was intentional, as our goal is focused on structure estimation rather than prediction, and BIC is a preferred choice in such cases.)

The full model, inclusive of both $\Delta p_{c}$ and $\Delta p_{p}$, demonstrated superior performance compared to the model excluding these variables. The BIC values for the full model were 16299 for Experiment 1 and 27756 in Experiment 2, outperforming the model without these variables (Experiment 1: BIC = 16819, Experiment 2: BIC = 28217). This outcome is presented in the manuscript as follows (l.559-566):
*“We ran a logistic regression on participants’ gambling choices with the foregoing eight regressors: EV, Risk,* $\Delta p_{c}$*,* $\Delta p_{p}$*, Prev. choices, Prev. reward, Prev. success, and Acc. reward (Fig 5; see Methods for details). This model showed the lowest value of BIC (Experiment 1: 16299, Experiment 2: 27756), outperforming the model only with EV and Risk (Experiment 1: 17094, Experiment 2: 29204), the model with EV, Risk,* $\Delta p_{c}$*, and* $\Delta p_{p}$ *(Experiment 1: 16663, Experiment 2: 28843), and the model with EV, Risk, Prev. choice, Prev. reward, Prev. success, and Acc. reward (Experiment 1: 16819, Experiment 2: 28217).”*

Moreover, several variables used in the regression exhibit high correlation (e.g., ∆𝑝𝑐 and EV, both of which incorporate the reward probability of the current option), resulting in multicollinearity. Addressing multicollinearity is crucial for accurately interpreting the effects of individual independent variables on participants' choices. To revise their modeling approach, the authors may find it beneficial to consult Wilson & Collins (2019) article on computational modeling of behavioral data.
**Response:** Thank you for this helpful advice. We have now included the full correlation matrix of the regressors used in our regression analysis (S11 Table). Although statistically significant, the correlations between $\Delta p_{c}$ and EV (Experiment 1: 0.24, Experiment 2: 0.31) were weak.

(l.1514) **S11 Table. Correlation structure and VIF of regressors entered into the main regression analysis.** ****p* < 0.001 (Bonferroni corrected).

Nevertheless, it is acknowledged that some regressors displayed moderate to strong correlations and high variance inflation factors. Despite the bootstrapped confidence intervals of the regression coefficients indicating a reasonable stability in their estimated values, we conducted several additional tests to explore the potential influence of multicollinearity. The results are presented in S12 Fig.

(l, 1517) S12 Fig. Stability of regression coefficient. Regression coefficients estimated for each experiment with the full model (white; identical to Fig. 5), full model with L1 regularization (blue), the model with only the primary regressors (EV, Risk, $\Delta p_{c}$, and $\Delta p_{p}$; pink), and the model excluding regressors with a VIF greater than five in either experiment (grey; see S11 Table for the values of VIF). Error bars represent 95% confidence bounds computed from bootstrap.

Specifically, we performed a rerun of the regression with L1 regularization, which robustly estimates regression coefficients even in the presence of multicollinearity. Additionally, we repeated the regression with only the primary regressors (excluding EV, Risk, $\Delta p_{c}$, and $\Delta p_{p}$) and ran it again, excluding regressors that exhibited a Variance Inflation Factor (VIF) greater than five in either experiment (regressors for EV, Risk, and Prev. success were excluded).

Overall, the outcomes of these analyses confirmed that the estimated values of regression coefficients remained reasonably stable. The result is presented in the manuscript as follows (l.608-630):

*“Several regressors in our analysis were correlated and had a high variance inflation factor (VIF; see S11 Table). Although the bootstrapped confidence intervals of the regression coefficients indicated a reasonable stability in their estimated values, we conducted several additional tests to investigate the potential influence of multicollinearity. Specifically, we reran the regression with L1 regularization, which robustly estimates regression coefficients in the presence of the multi-collinearity. In addition, we reran the regression with i) only the primary regressors (i.e., the regressors other than EV, Risk,* $\Delta p_{c}$*, and* $\Delta p_{p}$ *are excluded), and ii) excluding regressors that exhibited a VIF greater than five in either experiments (i.e., the regressors for EV, Risk, and Prev. success were excluded; S12 Fig). The estimated coefficients from both the L1 regularized regression and the regression with only the primary regressors showed no significant differences compared to those estimated from the original regression. (one-sample bootstrap test against the estimated value in the original model, two-tailed with Bonferroni correction, all p > 0.05). Significant differences were observed only in the regression that excluded regressors with high VIF. Specifically, the regression coefficient for* $\Delta p_{c}$ *was significantly different for both Experiment 1 and Experiment 2 (both p = 0.001), and that for* $\Delta p_{p}$ *was significantly different in Experiment 2 (p = 0.001). The elevated values of these regression coefficients are likely due to the removal of the EV regressor, which exerted the largest influence on participants' choices and showed positive association with* $\Delta p_{c}$ *and* $\Delta p_{p}$*. Nevertheless, the estimated values of the coefficients consistently exhibited the same signs in all regression analyses, and the overall results confirm a reasonable stability of the regression coefficients estimated from our original model.”*

Secondly, the regression analysis failed to demonstrate that accumulated reward predicted behavior in Experiment 2 (l.471-473). However, the effect of accumulated reward is an integral part of the authors' definition of risk-sensitive foraging theory (l.90-92). Consequently, without a clear definition of their model (as previously mentioned), it is premature to conclude that this theory explains the observed behavior in their experiment.
**Response:** We noticed that we had incorrectly encoded $\Delta p_{c}$ and $\Delta p_{p}$ regressors relative to the maximum reward probability in the current/past environment, which is not consistent with our hypothesis. We thus reanalyzed the data by coding the $\Delta p_{c}$ and $\Delta p_{p}$ regressors relative to the mean reward probability. **In this new regression model, the effect of accumulated reward was consistently significant in both experiments.**

(l. 573) **Fig 5. Regressors capturing the relative attractiveness of a gambling option compared to other options in the current and past environments play a significant role in explaining participants’ gambling propensity.** Participants’ gambling propensity was positively correlated with the deviation of reward probability of the current gambling option from the mean reward probability of all gambling options in the current environment ($\Delta p_{c}$). Participants’ gambling propensity was also positively correlated with the difference between the mean reward probability in the current and past environments ($\Delta p_{p}$). Additionally, participants’ gambling propensity was negatively correlated with the accumulated rearward (Acc. reward). Regressors with consistently significant regression coefficients across the two experiments are noted in bold. Error bars represent 95% confidence bounds computed from bootstrap. Two-tailed one-sample bootstrap tests against zero with Bonferroni correction were used for statistical tests of regression coefficients. **p* < 0.05; ***p* < 0.01.

Finally, another significant concern is the lack of discussion on how these findings relate to previous research in this area. Although the subject matter appears to fall within the broader field of context-dependent preferences (see, for example, Palminteri & Lebreton, 2021), no related literature is cited in the manuscript, particularly concerning the existing models proposed to explain this phenomenon (e.g., divisive or range-normalization models). It is essential for the authors to revise their Introduction and Discussion sections to incorporate references to the relevant literature. They should elucidate the differences between their proposed model and existing models, as well as demonstrate how their new model offers a superior explanation of behavior in this task compared to previous models.
**Response:** We thank reviewer for this helpful comment. We have now included citation to relevant literature in Introduction and Discussion, which includes the papers you suggested as well as others:

(Introduction, l.69-81) “*However, the framing of the decision problem [8,9], the history of prior outcomes [10,11], an individual's emotional state [12–16], hormone levels [17–19], observation of other’s choices [20], and aging [21] can alter the risk preference of a decision-maker. The foregoing observations show that the constancy of risk preference does not always hold [22]. Numerous studies have endeavored to elucidate the mechanistic underpinnings of changes in risk preference by employing diverse computational models with intricate utility functions tailored to specific contexts [23–28]. Notably, models with divisive normalization [24,27,29,30] , reference-point centering [26,31,32], and range adaptation [26,33,32] have prominently featured in this exploration. However, while these studies proficiently elucidate the mechanisms through which dynamic modulation of risk preference arises as a consequence of learning-based utility modulation, they lack a comprehensive articulation of the adaptive significance that underlies the specific structure of the utility function and its evolution over time.*

*”*

(Discussion, l.860-875) *“Second, participants were not informed about the environment that they will experience. Thus, they had to learn the reward statistics of the environment to adjust their choice behavior, indicating that participants have robust reward memories. However, we did not assess such learning effects as we refrained from constructing and testing specific computational models with intricate utility functions tailored to specific contexts aimed at providing a mechanistic account for the observed shift in risk preference during our experiment. As such, we did not explicitly test models incorporating a dynamic utility function. Noteworthy concepts in reinforcement learning and decision-making literature, such as the negative contrast effect [60], reference-point centering [26,31,32], range adaptation [26,33,32], and divisive normalization [24,27,29,30], are likely to provide mechanistic accounts to our findings. For example, the two environment-related regressors may be interpretable as range adaptions at two different time scales. The effects of environment-related regressors (*$\Delta p_{c}$ *and* $\Delta p_{p}$*) and their affect-based interpretation we offered shines light on the question why such adaptation occurs at the two different time scales. We hope that our findings lead to the exploration of computational models with adaptations at multiple time scales in explaining human economic behavior.”*

Citations added:
23. Koszegi B, Rabin M. A Model of Reference-Dependent Preferences.
24. Rangel A, Clithero JA. Value normalization in decision making: theory and evidence. Current Opinion in Neurobiology. 2012 Dec 1;22(6):970–81.
25. Louie K, Khaw MW, Glimcher PW. Normalization is a general neural mechanism for context-dependent decision making. Proceedings of the National Academy of Sciences. 2013 Apr 9;110(15):6139–44.
**26. Palminteri S, Lebreton M. Context-dependent outcome encoding in human reinforcement learning. Current Opinion in Behavioral Sciences. 2021 Oct 1;41:144–51.**
27. Glimcher PW, Tymula AA. Expected subjective value theory (ESVT): A representation of decision under risk and certainty. Journal of Economic Behavior & Organization. 2023 Mar 1;207:110–28.
28. Andrikogiannopoulou A, Papakonstantinou F. History-Dependent Risk Preferences: Evidence from Individual Choices and Implications for the Disposition Effect. The Review of Financial Studies. 2020 Aug 1;33(8):3674–718.
29. Carandini M, Heeger DJ. Normalization as a canonical neural computation. Nat Rev Neurosci. 2012 Jan;13(1):51–62.
30. Louie K, Glimcher PW. Efficient coding and the neural representation of value. Annals of the New York Academy of Sciences. 2012;1251(1):13–32.
31. Palminteri S, Khamassi M, Joffily M, Coricelli G. Contextual modulation of value signals in reward and punishment learning. Nat Commun. 2015 Aug 25;6(1):8096.
32. Bavard S, Lebreton M, Khamassi M, Coricelli G, Palminteri S. Reference-point centering and range-adaptation enhance human reinforcement learning at the cost of irrational preferences. Nat Commun. 2018 Oct 29;9(1):4503.
33. Bavard S, Rustichini A, Palminteri S. Two sides of the same coin: Beneficial and detrimental consequences of range adaptation in human reinforcement learning. Science Advances. 2021 Apr 2;7(14):eabe0340.

**Minor points :**

- It is unclear how many trials were conducted in each block. Clarification on this matter is needed:
  - In figure 1B, there are 60 trials in the poor and rich environments, and 36 trials in the intermediate environment (with reward magnitude varying from 10 to 65).
  - In line 586-590, the manuscript states that “in the poor, intermediate, and rich environments, reward probabilities were uniformly distributed with p = {0.2, 0.3, 0.4, 0.5, 0.6}, p = {0.4, 0.5, 0.6}, and p = {0.4, 0.5, 0.6, 0.7, 0.8}, respectively (Fig 1B, x-axis). The distribution of reward magnitude was the same for each reward probability and set as m = {15, 20, 25, ..., 65} (Fig 1B, y-axis).”
    This implies that there were 55 trials per block in the poor and rich environments, and 33 trials per block in the intermediate environment.
  - l.597-599: “The total number of trials during the rich and poor environments was 66, and that during the intermediate environment was 39”
- The total number of catch trials in each block is not specified.
  **Response:** We thank the reviewer for pointing out the above two points. We have added the following clarification about the total number of all trials as well as catch trials in “Behavioral task” section of Method as follows (l.986-998):
  *“Two types of catch trials were presented. One type had a gambling option with a reward magnitude of 10 and a reward probability below 1. Since the rejection of the gamble results in gain of 10 points, it is always better to reject the gamble in these trials. There were eight trials of this type in both the poor and rich environments, and three trials in the intermediate environment. The second type of catch trial had a gambling option with a reward probability of 1 and a reward magnitude above 10. Since the acceptance of the gamble results in a gain greater than 10 points, it is always better to accept the gamble in these trails. There were three trials of this type in each of the poor, rich and intermediate environments. Choice data of participants who responded incorrectly on more than 10% of the catch trials were excluded from the analysis (see exclusion criteria below). The total number of trials during the rich and poor environments was 66 (includes 11 catch trials), and that during the intermediate environment was 39 (includes 6 catch trials). “*
- Please provide information on the average number of failed trials due to slow response times.
  **Response:** Proportion of trials in which participants failed to respond were added in “Behavioral task” section of Method as follows (l.971-973):
  *“Overall, participants who met the inclusion criteria (see below) failed to respond on 0.37% of trials in Experiment 1 and 0.26% of trials in Experiment 2.”*

According to Figure 1B, the choices with a reward magnitude of 10 have been excluded from the analyses. However, this exclusion is not mentioned in the text. The rationale for removing these trials should also be explained.
**Response:** The trials with a reward magnitude of 10 were catch trials in which participants should always choose to reject if they understand the task rules correctly. Hence, they were removed from the analysis. This is now explicitly stated in the caption of Fig 1 as follows (l.184-185):
*“Gambles with a reward magnitude of 10 were excluded from the analysis as they were catch trials (see Method for details).”*

- The text mentions that “two groups of participants experienced different sequences of environment settings” in each experiment (l.600-601), but the number of participants per group is not specified. It is important to clarify this information (e.g., whether it was half of the participants).
  **Response:** This information was added to the “Behavioral task” section of Method between as follows (l.999-1008):
  *“We conducted two experiments. In each experiment, two groups of participants experienced different sequences of environment settings. In Experiment 1, one group experienced the poor environment followed by the rich environment (****Poor-Rich group or PR group, n = 108****), and the other group experienced the same environments in the reverse order (****Rich-Poor group or RP group, n = 136****) (Fig 1C, left). There was a self-paced rest between the first and second environments. In Experiment 2, one group experienced the poor environment between two intermediate environments (****Intermediate-Poor-Intermediate group or IPI group, n = 128****), and the other group experienced the rich environment between two intermediate environments (****Intermediate-Rich-Intermediate group or IRI group, n = 154****) (Fig 1C, right).”*
- The number of participants indicated in the method does not match the number of participants in the downloadable data files, both before and after applying exclusion criteria.
  **Response:** This is because data for those who did not complete the task are not included in the source datafiles stored in the GitHub repository. We explained this point in the “Exclusion criteria” section of Method as follows (l.1012-1021):
  *“A priori exclusion criteria were applied to ensure data quality [48]. Specifically, we excluded participants if they: (1) did not finish the task (Experiment 1, n = 1; experiment 2, n = 23); or (2) did not respond on at least 5% of the trials (n = 3, n = 7); or (3) made the same response on all the trails (n = 1, n = 0); or (4) made an error in at least 10% of the catch trials (n = 51, n = 88). Overall, 56 participants were excluded from experiment 1, and 118 participants were excluded from experiment 2. Consequently, our final sample comprised 244 participants in Experiment 1 and 282 participants in Experiment 2.* ***Note that data for those who did not complete the task (i.e., those who met exclusion criterion 1) are not included in source datafiles stored in the GitHub repository provided below.****”*
- Please report t-test results in the following format: Mean, Standard deviation, *t*(degrees of freedom) = *t*-value, *p* = *p*-value
  **Response:** We have made revisions to the manuscript accordingly. Information regarding Mean, SD, degrees of freedom, t-value, and p-value has been included either in the main text or supplementary tables where *t*-tests were applied.
- Can you provide statistical analyse supporting the following statements from the manuscript:
  - “The participants chose to gamble 3 frequently as the reward magnitude (x-axis of Fig 2A) and reward probability of the gamble (difference of the line types in Fig 2A) increased” (l.172-174)
    **Response:** We have provided information in S1 Fig and in its caption.

(l.1420) **S1 Fig. Participants inclination toward gambling exhibited a significant increase as the reward magnitude and reward probability increased.** Proportions of accepted gambles for various combinations of gambling parameters were computed across all participants in each experiment, and sigmoid curves were fitted using binomial regression. (A) Binomial regression results with reward magnitude as the explanatory variable showed a significantly positive slope for the sigmoid curve in both Experiment 1 (Left, slope = 0.044, 95% CI = [0.040, 0.049]) and Experiment 2 (Right, slope=0.041, bootstrapped 95% CI=[0.038, 0.045]). (B) Similarly, binomial regression results with reward probability as the explanatory variable demonstrated a significantly positive slope for the sigmoid curve in both Experiment 1 (Left, slope = 12.5, bootstrapped 95% CI=[11.4, 13.6]) and Experiment 2 (Right, slope = 12.1, 95% CI = [11.3, 13.1]).

- “In addition, we noticed that the difference between the IPI and IRI groups during the second block of experiment 2 is smaller than the difference between the PR and RP groups in the first block of experiment 1.” (l.257-259). Please also include a reference to Figure 2B.
  **Response:** This sentence has now been removed from the manuscript.
- “For experiment 1, the particle filter estimates for risk aversiveness were on average higher for the RP group than for the PR group” (l.270-271)
  **Response:** We have revised this section, but the corresponding information is now provided as follows (l.498-505):

*“Furthermore, in Experiment 1,* ***the mean risk aversiveness estimated by the particle filter was consistently higher for the RP group than for the PR group across the entire experiment (RP: M = 1.91×10^-2^, SD = 0.11×10^-2^, PR: M = 1.70×10^-2^, SD = 0.13×10^-2^ ;unpaired t-test, one-tailed; t(262) = 14.4, p < 0.001)****, in the poor environment (RP: M = 1.83×10^-2^, SD = 0.05×10^-2^, PR: M = 1.59×10^-2^, SD = 0.03×10^-2^; unpaired t-test, one-tailed; t(130) = 33.4, p < 0.001), and in the rich environment (RP: M = 2.00×10^-2^, SD = 0.07×10^-2^, PR: M = 1.82×10^-2^, SD = 0.06×10^-2^; unpaired t-test, one-tailed; t(130) = 15.0, p < 0.001).”*

- Figures 3C and 3D top right are not discussed in the text.
  **Response:** We revised the manuscript accordingly as follows (original Fig 3C and 3D is now plotted as Fig 4D, l.487-490):
  *“The risk-aversiveness estimated by the particle filter yielded quantitatively consistent results with the time-resolved analysis of the proportion of gambles accepted, highlighting the effects of the current environment (****Fig 4D top****) and past environments (Fig 4D bottom) on participants’ risk preference.”*
- Figures 3C and 3D right: the meaning of dots and horizontal lines is not mentioned in the legend.
  **Response:** The information is now provided in the caption of Fig 4 in the revised manuscript (l.431-432): *“Horizontal bars and unfilled dots represent the mean and raw values of the proportion of gambles accepted, respectively.”*
- Please provide clarification on the formulas used to compute ∆𝑝𝑐 and ∆𝑝𝑝. Were the formulas as follows?
  o ∆𝑝𝑐 = Reward probability of the current option – Maximum reward probability of the environment
  o ∆𝑝𝑝 = Maximum reward probability of the current environment – Maximum reward probability of the previous environment
- Can you specify how expected value (EV) and risk were computed?
  **Response:** We have added the definition of $\Delta p_{c}$ and $\Delta p_{p}$, EV and Risk to the manuscript in the “Logistic regression and simulation” section of Method as follows (l.1206-1216):
  *“To unravel the variables responsible for the pattern of shifts in the participants' risk preference observed in our experiments, we analyzed participants' choices with a logistic regression at the group level (Fig 4). Denoting the reward magnitude of a given gamble as* $m$ *and reward probability as* $p$*, we used the following regressors in our analysis: i) the expected value (****EV***$\boldsymbol{=mp}$*), ii) the risk of the gambling option* ***(Risk***$\boldsymbol{=}\boldsymbol{m}^{\boldsymbol{2}}\boldsymbol{p(1-p})$*), iii) the deviation of reward probability of the current option from the mean reward probability of all gambling options in the environment (*$\boldsymbol{\Delta}\boldsymbol{p}_{\boldsymbol{c}}\boldsymbol{=p-}\boldsymbol{p}_{\boldsymbol{c}_{\boldsymbol{mean}}}$*, where* $p_{c_{mean}}$ *represents the mean reward probability in the current environment),* iv) the difference between the mean reward probability in the current and past environments ($\boldsymbol{\Delta}\boldsymbol{p}_{\boldsymbol{p}}\boldsymbol{=}\boldsymbol{p}_{\boldsymbol{c}_{\boldsymbol{mean}}}\boldsymbol{-}\boldsymbol{p}_{\boldsymbol{p}_{\boldsymbol{mean}}}$, where $p_{p_{mean}}$ represents the mean reward probability in the previous environment), …*”*
- The link made between “the relative attractiveness of gambling options in the current environment compared to those in the past environments” and “an affect-based psychological mechanism” (l.474-478) is difficult to understand. Please provide clarification on this point.
  **Response:** We have now clarified this point as follows (l.783-796):
  *“To investigate whether the concept of accumulated reward in the risk-sensitive foraging formulation explains the effect of past environments on the risk preference in our experiments, we explicitly entered accumulated reward into our regression analysis to explain participants’ tendency to gamble. The risk-sensitive foraging theory predicts a negative regression coefficient for accumulated reward (Fig 4; Acc. reward). We found that the effect of accumulated reward was consistently negative across both experiments, as predicted. In addition, we found that the* $\Delta p_{p}$ *regressor (difference in the mean reward probability of the current and past environment), which captures the relative attractiveness of gambling options in the current environment compared to those in the past environments (i.e.,* $\Delta p_{p}$ *regressor captures how much better the gambling options in the current environment are compared to those in the past environment), was consistently positive and significant in both the experiments. We thus suggest that the effect of the past environment on participants’ risk preference was mediated via both the accumulated reward as well as an affect based psychological mechanism.”*

*”*

**Reviewer #3**

In this article, Mochizuki et al. show in two experiments that decisions under risk are influenced by the local context that they manipulated by changing the reward probability distribution. More specifically, participants were more risk averse during and after the rich environment compared to the poor environment.

I found the article well written and the experimental design neat. Yet, I have some concerns and suggestions which I hope will help improve the paper.
Thank
We extend our sincere thanks for conducting a comprehensive review of our manuscript and offering valuable insights. Your suggestions, particularly regarding the utilization of more intuitive analysis methods, have proven exceptionally beneficial, significantly enhancing the quality of the manuscript. In the following sections, we systematically address each of the concerns you raised.

At the conceptual level:
1) I am not quite sure why the authors framed their study with a distal mechanism (fitness maximisation as per foraging decision theory) to explain that the local context influences decisions. It is typically difficult to measure the equivalent in money to “the current physiological need” that would match the “environmental richness”. I found the discussion lines 485-500 rather indirect.

Other proximal theories like range adaptation or change in the (prospect theory utility function) reference point would also explain the results in a more direct way. For example, a reward probability of 0.6 is the best in the poor environment and may have the maximal value (as per range adaptation) or a positive utility (with a dynamical reference point) whereas it would have a low value or a negative utility in the rich environment and should be chosen less. This theoretical framework seems more in line with the regression analyses the authors performed (delta pp and delta pc).
**Response:** Thank you for emphasizing this crucial point. We recognize that mechanistic explanations, including range adaptation and dynamic reference point models, can offer a direct interpretation, particularly for the observed effect in the current environment. However, a model with only a single time constant might be insufficient in explaining the past environment effect. Hence, the novel insight provided by the observed past environment effect suggests a new dimension for models based on range adaptation and/or dynamic reference point.

In addition, our intention in framing the study within the context of fitness maximization, following risk-sensitive foraging theory, was not to delve into intricate mechanisms explaining the modulation of utility. Rather, our objective was to challenge the prevalent assumption in nearly all computational models of risk choice, namely utility maximization. Typically, these models assume that individuals seek more money, and what is considered "more" depends on the context. In contrast, our proposition is that individuals may choose to satisfy some internal thresholds even in the absence of explicit needs. While it is indeed possible to devise computational models based on this perspective, our approach here was to present descriptive findings without detailing such models. This choice allows our results to stand independently of the intricacies of computational models. By making this initial proposal, we believe our study contributes to the field by providing a broader account for further exploration within the scientific community.

We have discussed these issues in the introduction and discussion as follows:

Introduction (l.69-81): “*However, the framing of the decision problem [8,9], the history of prior outcomes [10,11], an individual's emotional state [12–16], hormone levels [17–19], observation of other’s choices [20], and aging [21] can alter the risk preference of a decision-maker. The foregoing observations show that the constancy of risk preference does not always hold [22]. Numerous studies have endeavored to elucidate the mechanistic underpinnings of changes in risk preference by employing diverse computational models with intricate utility functions tailored to specific contexts [23–28]. Notably, models with divisive normalization [24,27,29,30] , reference-point centering [26,31,32], and range adaptation [26,33,32] have prominently featured in this exploration. However, while these studies proficiently elucidate the mechanisms through which dynamic modulation of risk preference arises as a consequence of learning-based utility modulation, they lack a comprehensive articulation of the adaptive significance that underlies the specific structure of the utility function and its evolution over time.*”

Discussion (l.860-875): “*Second, participants were not informed about the environment that they will experience. Thus, they had to learn the reward statistics of the environment to adjust their choice behavior, indicating that participants have robust reward memories. However, we did not assess such learning effects as we refrained from constructing and testing specific computational models with intricate utility functions tailored to specific contexts aimed at providing a mechanistic account for the observed shift in risk preference during our experiment. As such, we did not explicitly test models incorporating a dynamic utility function. Noteworthy concepts in reinforcement learning and decision-making literature, such as the negative contrast effect [60], reference-point centering [26,31,32], range adaptation [26,33,32], and divisive normalization [24,27,29,30], are likely to provide mechanistic accounts to our findings. For example, the two environment-related regressors may be interpretable as range adaptions at two different time scales. The effects of environment-related regressors (*$\Delta p_{c}$ *and* $\Delta p_{p}$*) and their affect-based interpretation we offered shines light on the question why such adaptation occurs at the two different time scales. We hope that our findings lead to the exploration of computational models with adaptations at multiple time scales in explaining human economic behavior.*”

As you mentioned, a critical limitation here is that we could not directly show the equivalence in money of the current physiological need in animals’ energy intake. We discussed this point as follows (l.850-859):
“*Our current study has several limitations. First, while the fitness maximization framework correctly predicted the behavior of the participants in our experiments, a critical constraint is our inability to directly measure the participants' internal needs or thresholds which may drive their behavior. However, we note that in animal foraging, it is unlikely that animals adjust their behavior through a direct computation of energy requirements (need); rather, they likely rely on the feeling of hunger (affect). Similarly, we speculate that in our experiments, participants adjusted their choice propensity based on the subjective attractiveness of the option, rather than through a direct computation of need per se. This speculation is partly supported by the results of the regression analysis, but future studies are needed to further elucidate this aspect.*”

Regarding the design:
2) It seemed to me that participants were not instructed which environment they were in, which was confirmed in the last paragraphs of the discussion. I suggest to state it in Figure 1 caption and in the result section as well as in the methods.
**Response:** We thank you very much for spotting this important point. We have added this information as described below:
(Fig 1 caption, l.180-181) “*Note that participants were not informed about the presence of different environments.”*

(Result, l.167-169) “*In both experiments, participants were not given instructions regarding the presence of distinct environments or the sequence in which they would encounter them.”*

(Method, l.974-978) “*Participants experienced multiple blocks of risky choices during the main experiment. Between blocks, we manipulated the distribution of reward probability while keeping the distribution of reward magnitude fixed.* ***Thus, consecutive blocks had different mean reward probabilities, a detail that was not communicated to the participants during the experiment.***”

Regarding the model-agnostic results:
3) Is the decrease in gambling following the reach environment constant across the whole-session? I suggest the authors to show the model-agnostic temporal dynamics of gambling (potentially smoothed over a few trials). This would help see whether the change in risk attitude is immediate (unexpected) or progressive -and if so, the transition slope. This may be especially relevant to unpack figure 3B: the observed trend may result from a significant difference early in the last intermediate environment but not at the end due to adaption. I am aware of the attempt to model such dynamics, but model-agnostic data would be much more convincing.
**Response:** We thank reviewer for this very important suggestion. We computed moving average of proportion of gamble accepted as suggested and included it in the main part of the manuscript, Fig. 4A & B:

(l.416) **Fig 4. Dynamics in participants’ risk preference across the experiment.** (**A**) A moving average of the proportion of gambles accepted is computed for each group across Experiment 1 (left) and Experiment 2 (right). Each data point represents the proportion of gambles accepted by participants in a given trial, along with the two preceding and two succeeding trials, forming a window size of 5 trials. Vertical bars represent standard error in the proportion of gambles accepted. (**B**) The mean proportions of gambles accepted in each environment by each group. In both experiments, the mean proportion of gambles accepted were significantly higher when participants are experiencing the rich as compared to the poor environment (*p* < 0.001 for Experiment 1 and 2; top). In addition, the mean proportions of gambles accepted were significantly higher after participants experienced the poor than the rich environment (*p* < 0.001 for poor & rich environment in Experiment 1 and for the second intermediate environment in Experiment 2; bottom). One-tailed unpaired t-tests with Bonferroni correction were used for statistical comparisons of the proportion of gambles accepted (except for the comparison for the first intermediate in Experiment 2 where two-tailed test was employed given the initial prediction that there is no difference.) Horizontal bars and unfilled dots represent the mean and raw values of the proportion of gambles accepted, respectively.

This analysis affirmed the existence of both the current (Fig. 4B top) and past (Fig. 4B bottom) environment effects. As seen in Fig. 4A, participants' choice propensity swiftly adjusts to the new environment at the onset (current environment effect). However, it does not return to a fixed position for each environment (past environment effect).

4) In Experiment 2, I believe that the proportion of gambles accepted in the second environment could be compared to that of the first environment (i.e. rich 2 – intermediate versus poor 2 – intermediate). This sounds like a fair comparison and may help strengthen the otherwise edgy inference (p = 0.026 one tailed). This also applies to figure 3B where the results are reported as a trend.
**Response:** Thank you very much for suggesting this analysis. We have now included the figures for Experiment 2, where the proportion of the gambles accepted in the second block (S2 Fig) and third block (Fig. 3B, right) are compared after the subtraction of the first block (see figures in the response to the next comment).

5) Some key p-values are one-tailed and yet on the edge (e.g. figure 2B, p = 0.026). This may be improved following the previous suggestions. If not, I guess that it should be made explicit in the study limitations. Similarly, note that a trend for a p-value equals to 0.125 one-tailed is also a bit edgy (line 255).
**Response:** As you expected, we observed the predicted differences with greater significance after subtracting the behavior during the 1^st^ block (*p* < 0.001 and *p* = 0.006, for the comparison of S2 Fig and Fig. 3B, respectively).

- S2 Fig:

(l. 1432) **S2 Fig. IRI group exhibited a significantly greater decrease in proportion of gambles accepted compared to the IRI group in the second block.** Significance tests for the effect of environmental richness on gambling propensity. Change in the proportion of gambles accepted from the first to the second environment are computed for each group and compared (bootstrap test, one-tailed). Note that in the first environment both groups experienced the intermediate environment, and in the second block IPI and IRI group experienced the poor and rich environments, respectively. The shaded regions represent the variation, due to random samplings of participants, in the mean change in the proportion of gambles accepted as computed by bootstrap. The colored and black horizontal lines correspond to mean and 95% confidence intervals of the changes in the proportion of gambles accepted, respectively. ****p* < 0.001.

(Results, l. 259-266) “*In Experiment 2, to account for any differences in the initial risk tendencies and to quantify the effect of experiencing different environments on participants’ risk preferences, we revisited the foregoing comparison by subtracting the proportion of accepted gambles in the initial block where both groups encountered the intermediate environment. We verified that the IPI group exhibited a significantly greater increase in the proportion of gambles accepted in the poor environment compared to the IRI group in the rich environment (S2 Fig; p < 0.001; unpaired bootstrap test, one-tailed).*”

- Fig. 3B:

(l. 297) **Fig 3. Effects of the past environment on participants’ risk preference.** […] (**B**) In Experiment 2, while the IPI group accepted more gambles in the second intermediate environment than the IRI group, the direct comparison of the second intermediate environment did not reach statistical significance (left; bootstrap test, one-tailed). However, when comparing the change in the proportion of accepted gambles from the first to the second intermediate environment, the IRI group exhibited significantly larger downregulation in the proportion of gambles accepted than the IPI group (right; bootstrap test, one-tailed). The shaded regions represent the variation, due to random samplings of participants, in the mean of the proportion of gambles accepted as computed by bootstrap. The colored and black horizontal lines correspond to mean and 95% confidence intervals of the proportion of gambles accepted, respectively. **p* < 0.05; ***p* < 0.01; ****p* < 0.001.

(Results, l.317-328) “*In contrast, our hypothesis predicts that, while there will be downregulation in the proportion of gambles accepted during the final block as compared to the first block in both the groups (as the need will be lower than the initial need in both the groups), this downregulation will be small in the IPI group but much greater in the IRI group (because experiencing the rich environment in the second block will lower need more than experiencing the poor environment in the second block). While we found that the IPI group accepted more gambles than the IRI group during the final block, this difference did not reach significance (Fig 3B, right; p = 0.125, unpaired bootstrap test, one-tailed). However, as predicted, the downregulation in the proportion of gambles in the final block as compared to the first block was significantly greater in the IRI group compared to the IPI group (Fig 3B, left; p = 0.006; unpaired bootstrap test, one-tailed).*”

6) Still related to the statistics, I was surprised by the use of a fixed effect analysis (ignoring participants individual variance). Why not only using a more traditional random-effect for all the analyses a sin Figure S1 & 2 for all the data?
**Response:** We initially contemplated a participant-level analysis, as illustrated in Fig S1 and S2. However, technical challenges arose in implementing participant-level analysis for the particle-filter analysis (Fig. 3C and 3D), as detailed in the discussion (copy and pasted below). In addition, we conducted time-resolved analysis and included its results as you suggested, which can only be done at the group-level. To maintain consistency, we opted to report only group-level analysis in the main manuscript. We have reported participant-level analysis results in the supplementary sections.

(Discussion, l.903-913)*“Lastly, the main manuscript reports only group-level analysis, and participant level-analysis, wherever possible, is relegated to the supplementary sections of the manuscript. This is partly due to the high noise associated with the particle-filter estimation of the risk parameter in the state-space model (Fig 4C & D), making it impossible to reliably estimate the change in risk preference of a single participant under the current experimental settings. This is most likely because the noise associated with the observation process (the sigmoid choice model) is too high. In addition, the computational cost of the particle filter algorithm was very high, such that a single optimization procedure took about six hours. Additionally, the time-resolved analysis (Fig. 4A, B & 6) could only be done at the group-level. To maintain consistency, we opted to report only group-level analysis in the main manuscript.”*

We also note here that in our experiment, participants encounter identical sets of gambles in each environment. Thus, both participant-level analysis and group-level analysis theoretically yield identical results when examining the proportions of accepted gambles, as depicted in Fig 2B, 3A, and 3B. This equivalence is evident when comparing the mean proportion of accepted gambles obtained through the proportion derived from group-level analysis

$$p_{group}=\frac{\sum_{i} m_{i}}{n*m_{all}},$$

with mean proportion derived from participant-level analysis

$$p_{particioant}=\frac{1}{n}\sum_{i} \frac{m_{i}}{m_{all}}=\frac{\sum_{i} m_{i}}{n*m_{all}},$$

where *n* is the number of participants in each group, $m_{i}$ is the number of times *i-*th participant chose to gamble in each environment, and $m_{all}$ is the number of gambles (or trials) in that environment. Only if participants do not respond to some trials, the value for $m_{all}$ can vary, but the proportion of no response was very small as indicated in l.933 (0.37% of trials in Experiment 1 and 0.26% in Experiment 2).

These points are explained in Method of the revised manuscript (l.1048-1057):
“*In the supplementary analysis, we computed the q for each participant (Figs S3 and S4). Note that the group-level analysis (main manuscript) and the participant-level analysis (supplementary materials) of the proportion of gambles accepted should return the same results if participants respond on all the trials. Given that participants responded on almost all the trials (failure to respond rates were 0.37% in Experiment 1 and 0.26% in Experiment 2) and to maintain methodological consistency with the analysis of the proportion of gambles accepted across trials and of the risk-aversiveness employing the particle-filter (Figs 4; details provided below and in Discussion), in the main manuscript we chose to present the primary analysis of the proportion of gambles accepted at the group-level (Figs 2B & 3).*”

Regarding the model-based analyses:
7) The current particle filter model lacks of goodness of fit metrics (e.g. pseudo-r2 and balanced accuracy) and validation by e.g. overlapping the model predictions with the actual data. It is rather unclear whether the changes in alpha presented in figure 3 C & D are, if even impactful, to the same magnitude order as in the choice data.
**Response:** We thank the reviewer for this useful suggestion. We now show that the risk-aversiveness estimated by the particle filter was highly correlated (l.458; (Experiment 1: r = -0.91, p < 0.001; Experiment 2: r = -0.77, p < 0.001) with the proportion of gambles accepted computed across the experiments. We have also simulated the choices from the estimated risk-aversiveness, and overlayed it with participants’ choices (S10 Fig). The resulting mean proportion of the gambles accepted confirmed the presence of the current and past environment effect observed the real data (Fig. 4A & B).

(l.1497) **S10 Fig. Dynamics in simulated choice generated from risk aversiveness estimated by the particle filter.** (**A**) A moving average of the proportion of gambles accepted is computed for the simulated choices of each group (*n* = 120) across Experiment 1 (left) and Experiment 2 (right). Each data point represents the proportion of gambles accepted in current trial and the preceding and succeeding two trials, forming a window size of 5 trials. Error bars represent standard errors. (**B**) Mean proportion of gambles accepted in each environment by each group. In both experiments, simulation showed that the proportion of the gambles accepted is significantly higher during the rich as compared to the poor environment (*p* < 0.001 for Experiment 1 and 2; top). In addition, the proportion of the gambles accepted was significantly higher after experiencing the poor rather than the rich environment (*p* < 0.001 for poor & rich environment in Experiment 1 and for the second intermediate environment in Experiment 2; bottom). One-tailed unpaired t-tests with Bonferroni correction were used for statistical comparisons of proportion of gambles accepted (except for the comparison for the first intermediate in Experiment 2 where two-tailed test was employed given the initial prediction that there is no difference.) ****p* < 0.001.

8) Was this model used on all trials or only the intermediate ones?
**Response:** As in the rest of the manuscript, only those trials with reward probability *p* = {0.4, 0.5, 0.6} are analyzed. We clarified this point in Method (l.1195-1198) as follows:
“For unanalyzed trials (see Fig 1) and trials with no response, the participants’ choices were treated as missing data *(i.e., for all environments, we only used trials with reward probability p = {0.4, 0.5, 0.6} and with participants' response, as in the rest of the analysis)*.”

9) Risk averseness simulations suggest that there is no difference in Experiment 1 between rich and poor environment in the second block. Yet, it seems not to be the case looking at Figure 3A (i.e. there seems to be a significant difference). Can the authors report the statistics for that comparison in both the data and the simulations? If the simulation fails to capture this effect, it would invalidate it. If there is no model-agnostic differences, then this may be problematic for the theory overall.
**Response:** We apologize for any confusion caused. In the original manuscript, Fig. 3A was plotted with varying ranges on the y-axis, making them hard to compare visually. In response to the comment from Reviewer 1, this has been rectified in the revised manuscript, as shown below in Fig. 3A (copy and pasted). In the revised figure, it can now clearly be seen that the proportion of gambles accepted in the poor environment by the RP group (middle, right blob) and that in the rich environment by the PR group (left, left blob) are overlapping.

(l.297) **Fig 3. Effects of the past environment on participants’ risk preference.** (**A**) In Experiment 1, significantly more gambles were accepted when participants experienced the poor environment before the rich environment. The PR group accepted significantly more gambles than the RP group (left), in both the poor (middle) and rich (right) environments (bootstrap test, one-tailed). […] The shaded regions represent the variation, due to random samplings of participants, in the mean of the proportion of gambles accepted as computed by bootstrap. The colored and black horizontal lines correspond to mean and 95% confidence intervals of the proportion of gambles accepted, respectively. **p* < 0.05; ***p* < 0.01; ****p* < 0.001.

As you mentioned, the same trend (that is no significant difference the risk-aversiveness between two groups in the second block of Experiment 1) is observed in the risk-aversiveness estimated by the particle filter (Fig 4C left & D bottom left):

(l. 416) **Fig 4. Dynamics in participants’ risk preference across the experiment.** […] (**C**) Risk aversiveness $\alpha$ of each group estimated by a particle filter. Shaded regions are 90% confidence intervals for risk aversiveness $\alpha$. (D) Risk aversiveness $\alpha$ estimated by the particle filter is plotted mirroring the format of (B). In both experiments, participants were significantly more risk averse when experiencing the rich as compared to the poor environment (*p* < 0.001 for Experiment 1 and 2; top). In addition, participants were significantly more risk averse after experiencing the rich rather than the poor environment (*p* < 0.001 for the poor & rich environment in Experiment 1 and for the second intermediate environment in Experiment 2; bottom). ****p* < 0.001.

10) Similarly, the model predicts a significant difference (please report the stats) in the first intermediate environment of Experiment 2. This was not observed in the data and is therefore problematic.
**Response:** The discrepancy in risk propensity between the two groups in the initial block of Experiment 2 is evident both in the risk aversiveness estimated by the particle filter (*p* < 0.001; Fig 4B) and in the mean proportion of accepted gambles in the time-resolved analysis (*p* < 0.001; Fig 4A). Therefore, the outcomes align consistently in both the state-space model analysis and the model-free analysis. This alignment is further supported by the enhanced significance observed in the comparisons of the second and third blocks after subtracting the first block, as per your suggestion.

However, this initial difference between two groups in Experiment 2 was unexpected, and we commented on this in our discussion (l.896-902):

*“Sixth, in Experiment 2, the two groups of participants showed a significant difference in the proportion of gambles accepted during the first block, in which both groups experienced the intermediate environment. Thus, direct comparisons of interest for the effect of the current (Fig 2B; p = 0.026) and past environments (Fig 3A; p = 0.125) were much weaker than anticipated. However, after subtracting the performance during the initial block as baseline performance, the comparisons of interest were robust for both the effects (current environment: S2 Fig, p < 0.001; past environment: Fig 3B, p = 0.006).”*

Additionally, it is important to emphasize that our aim in employing the state-space models is to offer a descriptive account of the dynamics in participants' risk propensity, rather than presenting a mechanistic explanation of how choices are generated. This perspective has been discussed in the Discussion section (see below, l.803-820), and the relevant portion of the Method section has been revised to enhance clarity (see l.1147-1204).

(Discussion, l.803-820) “*Behavioral economics and recent decision making literature investigate how human decision biases occur by developing computational models that explain observed behavior better than classical theories [6,47–49,23,26,24,29,27]. However, there is no consensus regarding why these biases occur in human economic decision-making [1,50]. To tackle this issue, our primary focus in data analysis centered on descriptive statistics (see Figs 2, 3, and 4A & B) rather than constructing and testing specific computational models aimed at providing a mechanistic account for the observed shift in risk preference during our experiment. We complemented this approach with a computational model with minimal assumptions. Specifically, we employed the risk-return model to encapsulate participants' risk aversion with a single model parameter within the utility function, and estimated its dynamic transition using a particle filter, which relies solely on the assumption of the Markov property in the temporal transition of the risk aversion parameter (see Figs 4C & D). This analytical strategy was deliberately chosen to contrast with the conventional method of analysis, wherein computational models are intricately detailed for the construction of utility functions and its dynamics [23–27]. In doing so, we aimed to provide a unique perspective by adopting an approach which allows us to test the qualitative predictions made by the frameworks of fitness and utility maximization, incorporating minimal assumptions in our computational accounts.*”

11) I suggest the authors to start to fit a static prospect theory model (perhaps for each environment specifically versus altogether and see which model wins) to the data so as to get a sense of how this basic model captures the data (how does risk aversion change across environment).
**Response:** As response to your comment and other reviewers’ comments, we now included the results of prospect theory-based models, using both the multiplicative model ($U=v\left( m \right)\pi(p)$) as well as the additive model ($U=\left( 1-w_{p} \right)v\left( m \right)+w_{p}\pi(p)$). The results is presented in the newly added section “Fitting of computational models confirms the presence of the current and past environment effects (l. 331-405)” in Results and the definition of the models and the fitting method are detailed in “Computational models and their fitting (l.1086-1135)” in Methods.

The results did not give simple interpretation (l. 373-380):
*“We tested our predictions (one-tailed t-tests in the direction of our prediction with Bonferroni correction) regarding the changes in risk-preference for each model parameter of the best performing models separately (i.e.,* $\alpha$ *for the risk-return model with,* $\lambda$ *and* $\gamma$ *for the multiplicative model,* $\lambda$*,* $\gamma$ *and* $w_{p}$ *for the additive model; S7 Table). The predicted changes in risk-preference parameters were significant for the risk-aversiveness* $\alpha$ *of the risk-return model, and the risk-proneness* $\gamma$ *of the additive model, consistently across Experiment 1 and Experiment 2. This result underscores the influence of both the current and past environmental effects on participants' risk preferences.”*

This point is noted in Discussion as a weakness of this study (l. 876-885):
“*Third, related to the previous caveat, our results of model fitting (S5 Table, S6 Fig, S7 & 8 Tables) were inconclusive. The effects of the current and past environments were mediated by different risk parameters depending on the choice of the utility model (the risk-return model, and prospect theory-based multiplicative and additive models) and the choice rule (the sigmoid choice rule and the approach-avoidance choice rule). We also note that the prospect theory-based models might not be entirely suitable for the current experiments due to the constrained range of reward probabilities. Despite this, we hope that the robust observation of the current and past environment effects in our model-free analysis will prompt future studies to delve deeper into the computational underpinnings behind the observed modulations in risk preferences.*”

Then different dynamics could be tested, for example the outcome could be normalised with time (due to range adaptation) or the reference point could evolve according to the recent past (something than could be captured with a decay over past outcomes e.g. as in Andrikogiannopoulou & Papakonstantinou 2019) without the need for a change in risk aversion. Parameters and model recovery could then be performed, and model comparison would indicate which of those models best explain the data. This model could then be validated against the data.
**Response:** As previously discussed, our anticipation is that a model featuring only a single time constant lacks adequacy in elucidating the past environment effect we observed. Additionally, we deliberately refrain from introducing a new computational model to account for the past environment effect. This decision aligns with our intention to maintain a distinct stance from studies that delve into mechanistic explanations for risk choices. We have incorporated relevant text into the introduction and discussion sections of the manuscript (refer to response to comment (1) of reviewer 3 for details).

The suggested reference (Andrikogiannopoulou & Papakonstantinou 2020) has been added to the manuscript (ref [28], l.1318).

12) The authors attributed changes in decision (e.g., whether to gamble) to risk preference changes. Recent literature in the gain domain shows increased gambling behaviour may be attributed to Pavlovian approach motivation, but not risk attitudes (Rutledge [et.al](http://et.al/)., Current Biology, 2016). Could they test this hypothesis?
**Response:** We thank you for this suggestion. We fitted the approach-avoidance model to our data. However, the result was in conclusive that the inclusion of the parameter $\eta$ that captures Pavlovian approach motivation resulted in the decrease in the information criterion in the best performing model but increased for other models (S5 Table). Furthermore, the current and past environment effects were observed in some model but not for others. We reported the results as follows in Results (l.381-405):
*“Additionally, we also fitted the foregoing models by replacing the sigmoid choice rule with an approach-avoidance choice rule (see Methods). In these models, the risk-proneness parameter* $\eta$ *biases the probability of gambling by changing the maximum (if* $\eta<0$*) or minimum (if* $\eta>0$*) probabilities to gamble in the sigmoid curve. The parameter* $\eta$ *serves as a risk-proneness parameter, and, consequently, we predicted it to be greater during and after experiencing the poor environment compared to the rich environment.*

*The model comparison of the approach-avoidance version of the models with iBIC showed that the best model was the additive model including both* $\lambda$ *and* $\gamma$ *(S8 Table). Within the multiplicative models, the best model was also the one including both* $\lambda$ *and* $\gamma$*. Among the models with three parameters, the risk-return model (that includes* $\alpha$*,* $\beta$*, and* $\eta$*) was the best model. Lastly, model comparison between the approach-avoidance choice-based models and sigmoid choice-based models were inconclusive, such that with the extra model parameter* $\eta$*, iBIC improved for the risk-return model, but not for the multiplicative and additive models.*

*We tested our predictions (one-tailed t-tests in the direction of our prediction with Bonferroni correction) regarding the changes in risk-preference for each model parameter of the best performing models separately (i.e.,* $\alpha$ *and* $\eta$ *for the risk-return model,* $\lambda$*,* $\gamma$ *and* $\eta$ *for the multiplicative model,* $\lambda$*,* $\gamma$*,* $w_{p}$ *and* $\eta$ *for the additive model; S8 Table). The predicted changes in risk-preference parameters were significant for the risk-proneness* $\eta$ *of the risk-return model, and the risk-proneness* $\gamma$ *of the multiplicative model, and the risk-proneness* $\eta$ *of the additive model, consistently across Experiment 1 and Experiment 2. This result confirms again the influence of both the current and past environmental effects on participants' risk preferences.”*

This is reported as a limitation of our study in Discussion (l.876-885):
“*Third, related to the previous caveat, our results of model fitting (S5 Table, S6 Fig, S7 & 8 Tables) were inconclusive. The effects of the current and past environments were mediated by different risk parameters depending on the choice of the utility model (the risk-return model, and prospect theory-based multiplicative and additive models) and the choice rule (the sigmoid choice rule and the approach-avoidance choice rule). We also note that the prospect theory-based models might not be entirely suitable for the current experiments due to the constrained range of reward probabilities. Despite this, we hope that the robust observation of the current and past environment effects in our model-free analysis will prompt future studies to delve deeper into the computational underpinnings behind the observed modulations in risk preferences.*”

13) The regression analysis is capturing the range adaptation idea (without being a structural model) with delta pp and delta pc. Yet, these two regressors may be equivalent to static dummy variables. I suggest the authors to try to equip them with a temporal dynamics that would account for the temporal dynamics that may exist in the data.
**Response:** We note that the $\Delta p_{c}$ regressor is temporally dynamic, in that its value changing on a trial-by-trial basis. In addition, we also note that $\Delta p_{p}$ is static within a block but dynamic across blocks, and encodes the difference between two consecutive environments, aligning with our hypotheses. In addressing your comment and Reviewer 2’s feedback, we have clarified its definition in the Methods (l.1206-1216):  *“To unravel the variables responsible for the pattern of shifts in the participants' risk preference observed in our experiments, we analyzed participants' choices with a logistic regression at the group level (Fig 4). Denoting the reward magnitude of a given gamble as* $m$ *and reward probability as* $p$*, […], iii)* ***the deviation of reward probability of the current option from the mean reward probability of all gambling options in the environment (***$\boldsymbol{\Delta}\boldsymbol{p}_{\boldsymbol{c}}\boldsymbol{=p-}\boldsymbol{p}_{\boldsymbol{c}_{\boldsymbol{mean}}}$***, where*** $\boldsymbol{p}_{\boldsymbol{c}_{\boldsymbol{mean}}}$ ***represents the mean reward probability in the current environment),* iv) the difference between the mean reward probability in the current and past environments (**$\boldsymbol{\Delta}\boldsymbol{p}_{\boldsymbol{p}}\boldsymbol{=}\boldsymbol{p}_{\boldsymbol{c}_{\boldsymbol{mean}}}\boldsymbol{-}\boldsymbol{p}_{\boldsymbol{p}_{\boldsymbol{mean}}}$**, where** $\boldsymbol{p}_{\boldsymbol{p}_{\boldsymbol{mean}}}$ **represents the mean reward probability in the previous environment)**, …*”*

14) Can the authors report the goodness of fit of the regression analysis and overlay the model prediction with the actual data? **Response:** We have provided BIC values of nested regression models in Resutls as follows (l.559-566):
*“We ran a logistic regression on participants’ gambling choices with the foregoing eight regressors: EV, Risk,* $\Delta p_{c}$*,* $\Delta p_{p}$*, Prev. choices, Prev. reward, Prev. success, and Acc. reward (Fig 5; see Methods for details). This model showed the lowest value of BIC (Experiment 1: 16299, Experiment 2: 27756), outperforming the model only with EV and Risk (Experiment 1: 17094, Experiment 2: 29204), the model with EV, Risk,* $\Delta p_{c}$*, and* $\Delta p_{p}$ *(Experiment 1: 16663, Experiment 2: 28843), and the model with EV, Risk, Prev. choice, Prev. reward, Prev. success, and Acc. reward (Experiment 1: 16819, Experiment 2: 28217).”*

We have also simulated choices with the estimated regression coefficient, and overlayed it with participants’ choices (Fig 6, copy and pasted below). The resulting mean proportion of the gambles accepted confirmed the presence of the current and past environment effect observed in the real data (Fig. 4A & B):

(l.671) **Fig 6. Environment-related regressors are necessary in recovering the observed dynamics in risk preference.** (**A, B**) Regressors shown in bold in Fig 5 and their coefficients were used to simulate participants’ gambling choices (*n* = 120 for each group). The moving average of the proportion of gambles accepted for each trial (A) and their environment-wise averages (B) were calculated for each group. The resulting dynamics showed close similarities with that estimated from the real data (Figs 4A), recovering the effect of the current (B top) and past (B bottom) environments. (**C, D**) The dynamics of the proportion of gambles accepted present in the real data could not be recovered when the environment-related regressors $\Delta p_{c}$ and $\Delta p_{p}$ are excluded (the regressors shown in bold in S13 Fig and their coefficients were used to simulate participants’ gambling choices; *n* = 120 for each group). (**A, C**) Error bars represent standard errors. Shaded regions are 95% confidence intervals of the proportion of gambles accepted computed for the real data. (**B, D**) Horizontal bars and unfilled dots represent the mean and raw values of the proportion of gambles accepted, respectively. One-tailed unpaired t-tests with Bonferroni correction were used for statistical comparisons of the mean proportion of gambles accepted (except for the comparison for the first intermediate in Experiment 2 where a two-tailed test was employed given the initial prediction that there is no difference.) ****p* < 0.001.

15) Can the authors report the results of this regression when not controlling for previous choice, previous reward, previous success, and accumulated reward?
**Response:** We have provided BIC value of a regression models without previous choice, previous reward, previous success, and accumulated reward, as commented above in the response to your comment (14). Inclusion of these variables improved the model performance.

16) Would it be possible that the model without the normalisation terms (i.e. the deltas) would be able to capture the data if it is fit to the data? At the moment, as far as I understood, the model without the deltas is not fit to the data (instead, the estimates of the full model are used, without the deltas as generative parameters).
**Response:** In response to this comment and Reviewer 2’s comment we have also simulated choices for the model without environment-related regressors $\Delta p_{c}$ and $\Delta p_{p}$, where the regression coefficients estimated without them were used (Fig. 6, copy and pasted above in response to your comment (14)). The resulting mean proportion of the gambles accepted did not recover the current and past environment effect observed in the real data (Fig. 4A & B). This is explained in the manuscript as follows (l.690-701):
“*In contrast, in simulation 2, where the environment-related regressors* $\Delta p_{c}$ *and* $\Delta p_{p}$ *were excluded (Fig 6C & D), the observed proportion of gambles accepted were negatively correlated with that observed in the real choices (Experiment 1: r = -0.25, p < 0.001; Experiment 2: r = -0.35, p < 0.001). Furthermore, we did not observe qualitative consistency between the mean proportion of gambles accepted computed for the simulated choices and the real choices both for Experiment 1 and Experiment 2, failing to recover the effects of the current environment (Fig 6D, top) and past environments (Fig 6D, bottom) . In particular, the mean proportion of gambles accepted were higher during the rich than during the poor environment in the first block of Experiment 1 (poor: M = 0.54, SD = 0.03, rich: M = 0.57, SD = 0.04) and in the second block of Experiment 2 (poor: M = 0.54, SD = 0.03, rich: M = 0.60, SD = 0.03), contrary to our prediction and the trends observed in the real data (Figs 4A and B).*”

17) I liked the attempt to assess the effect of the deltas in the simulation by removing them. Yet, I suggest to directly compare the models to the actual data rather than indirectly to the potentially dubious simulated particle filter model (see my comments above). I do suggest to investigate the respective effect of each as well as their additive effect. I noticed the correlations figure 5 C & D, but I think that a more direct mapping between the model and the data would be more informative.
**Response:** We thank reviewer for this suggestion too. We performed the suggested analysis in S14 Fig by dropping each of $\Delta p_{c}$, $\Delta p_{p}$, Prev. choice, and Acc. reward regressors in turn. We simulated choice from each model and overlayed the resulting proportion of gambles accepted with that calculated for the real data. The results suggest that $\Delta p_{c}$ is responsible for the current environment effect, while $\Delta p_{p}$ is responsible for the past environment effect:

(l.1532) **S14 Fig.** $\boldsymbol{\Delta}\boldsymbol{p}_{\boldsymbol{c}}$ **and** $\boldsymbol{\Delta}\boldsymbol{p}_{\boldsymbol{p}}$ **are necessary for recovering the current and past environment effects, respectively.** (**A, B**) The simulation results of the model excluding $\Delta p_{c}$ (*n* = 120 for each group). The moving average of the proportion of gambles accepted for each trial (A) and their environment-wise averages (B) were calculated for each group. (**C, D**) Mirroring the format of A & B but plotted for the model excluding $\Delta p_{p}$. (**E, F**) Mirroring the format of A & B but plotted for the model excluding Prev. choice. (**G, H**) Mirroring the format of A & B but plotted for the model excluding Acc. reward. (**A, C, E, G**) Error bars represent standard errors. Shaded regions are 95% confidence intervals of the proportion of gambles accepted computed for the real data. (**B, D, F, H**) Horizontal bars and unfilled dots represent the mean and raw values of the proportion of gambles accepted, respectively. One-tailed unpaired t-tests with Bonferroni correction were used for statistical comparisons of the mean proportion of gambles accepted (except for the comparison for the first intermediate in Experiment 2 where two-tailed test was employed given the initial prediction that there is no difference.) **p* < 0.05; ****p* < 0.001.

The results are explained in Results as follows (l.802-711):
“*To further assess the necessity of the environment-related regressors in explaining the dynamics in participants’ risk preferences, we generated simulated choices by systematically excluding each of the following regressors from the original model in separate iterations:* $\Delta p_{c}$*,* $\Delta p_{p}$*, Prev. choice, and Acc. reward (S14 Fig). In the model where* $\Delta p_{c}$ *was excluded (S 14 Fig A & B), the impact of the current environment deviated from the expected pattern (S14 Fig B, top), while the influence of the past environment aligned with the observed dynamics in the real choices (S14 Fig B, bottom). This observation supports the notion that* $\Delta p_{c}$*, which reflects the attractiveness of an option relative to others in the local environment, plays a critical role in explaining the effect related to the current environment.*”

In fact, if the normalisation terms (i.e. the deltas) with a temporal components (e.g. the reference point sensitivity to the recent outcomes) are plugged to a PT model, the fit of that model could be overlaid to the temporal model-agnostic data.
**Response:** Thank you for this suggestion. However, as discussed earlier, for the purpose of the current study, we would like to refrain from detailed modeling approach to make our claim clear. Please see response to your comment (1) for details on the text added to the main manuscript.
